# The KDM6B/SLC10A2 Axis Suppresses MDSCs Recruitment via ERK/AP‐1 Signaling in Colorectal Cancer

**DOI:** 10.1002/advs.202514086

**Published:** 2025-12-12

**Authors:** Zhibo Hu, Jing Xun, Bin Liu, Xiaolin Jiang, Yingdi Han, Huichao Yang, Qi Gao, Ruifang Gao, Aimin Zhang, Xueliang Wu, Dapeng Zhang, Dihua Li, Tian Li, Shimin Yang, Ximo Wang, Xiangyang Yu, Qi Zhang

**Affiliations:** ^1^ Tianjin Nankai Hospital Tianjin Medical University; Institute of Integrative Medicine for Acute Abdominal Diseases Tianjin Key Laboratory of Acute Abdomen Disease Associated Organ Injury and ITCWM Repair Tianjin 300100 China; ^2^ Department of Gastric Surgery, Tianjin Medical University Cancer Institute & Hospital, National Clinical Research Center for Cancer; Tianjin Key Laboratory of Digestive Cancer Tianjin's Clinical Research Center for Cancer Tianjin 300060 China; ^3^ Tianjin Institute of Medical and Pharmaceutical Sciences Tianjin 300020 China; ^4^ Tianjin Nankai Hospital Tianjin Medical University Tianjin 300100 China; ^5^ The First Affiliated Hospital of Hebei North University Hebei 075000 China; ^6^ Tianjin Medical University Institute of Integrative Medicine for Acute Abdominal Diseases Tianjin Key Laboratory of Acute Abdomen Disease Associated Organ Injury and ITCWM Repair Tianjin 300100 China

**Keywords:** colorectal cancer, immunosuppressive, lysine‐specific demethylase 6B (KDM6B), myeloid‐derived suppressor cells (MDSCs), solute carrier family 10 member 2 (SLC10A2)

## Abstract

Colorectal cancer (CRC) progression is regulated by an immunosuppressive tumor microenvironment, but the epigenetic mechanisms governing this milieu remain unclear. This study identifies the histone demethylase KDM6B as a key regulator of myeloid‐derived suppressor cells (MDSCs) recruitment in CRC. Intestinal epithelial‐specific KDM6B deletion promotes tumor growth by increasing MDSCs‐mediated immunosuppression. Mechanistically, KDM6B directly transcriptionally activates solute carrier family 10 member 2 (SLC10A2), whereas its loss increased H3K27me3 repression at the SLC10A2 promoter, activating the ERK/AP‐1 pathway and subsequent CXCL/CXCR2‐dependent MDSC recruitment. Clinically, KDM6B expression positively correlated with SLC10A2 levels and inversely correlated with MDSC infiltration in human CRC specimens. More importantly, KDM6B knockdown conferred resistance to anti‐PD‐1 therapy in CRC, whereas its overexpression synergized with anti‐PD‐1 therapy. In conclusion, this study establishes the KDM6B–SLC10A2 axis as a novel epigenetic immune checkpoint, highlighting its potential as a therapeutic target for reprogramming the immunosuppressive microenvironment in CRC.

## Graphical abstract

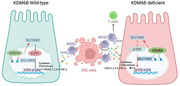

## 1 Introduction

Colorectal cancer (CRC) remains a leading cause of cancer‐related mortality worldwide, with a significant proportion of patients presenting at advanced stages and exhibiting poor survival outcomes.^[^ ^1^ ^]^ Despite the remarkable success of immune checkpoint blockade (ICB) in certain malignancies, its efficacy in CRC is largely restricted to tumors with microsatellite instability‐high (MSI‐H) or mismatch repair‐deficient (dMMR) phenotypes.^[^ ^2^ ^]^ The vast majority of microsatellite‐stable (MSS) tumors—accounting for 80–85% of CRCs—are refractory to current immunotherapies, highlighting the critical need to elucidate the molecular mechanisms underlying immune evasion in these tumors.^[^ ^3^ ^]^

Epigenetic dysregulation has emerged as a central orchestrator of both carcinogenesis and immune escape, creating synergistic opportunities for the combination of epigenetic therapy and immunotherapy. In CRC, such epigenetic aberrations—encompassing DNA methylation, histone modifications, and noncoding RNA dysregulation—drive tumor initiation, invasion, and metastasis by reshaping the transcriptional landscape of intestinal epithelial cells.^[^ ^4^ ^]^ Lysine‐specific demethylase 6B (KDM6B), also known as jumonji domain‐containing protein D3 (JMJD3), is a demethylase that specifically demethylates H3K27me2/3 in gene promoters to promote gene expression. Studies have shown that KDM6B is critical for development, inflammation, and cancer in a context‐dependent manner.^[^ ^5^, ^6^ ^]^ In breast cancer, KDM6B can mediate NSUN2 to increase osteoclast differentiation and promote bone metastasis in breast cancer,^[^ ^7^ ^]^ whereas in GBM, inhibition of KDM6B in myeloid cells increases antigen presentation and overcomes immune checkpoint resistance by suppressing immunosuppressive mediators (Mafb/Socs3/Sirpa).^[^ ^8^ ^]^ Thus, KDM6B may serve as a critical nexus bridging epigenetic regulation and inflammatory responses. These studies suggest that KDM6B may play a critical role in modulating the immunosuppressive tumor microenvironment.

Myeloid‐derived suppressor cells (MDSCs) represent a heterogeneous population of pathologically activated immature myeloid cells that play a pivotal role in tumor immune evasion.^[^ ^9^ ^]^ These cells are generally divided into two major subsets—polymorphonuclear MDSCs (PMN‐MDSCs) and monocytic MDSCs (M‐MDSCs)—that exert immunosuppressive effects through distinct mechanisms.^[^ ^10^ ^]^ MDSCs mediate potent suppression of antitumor immunity via multiple pathways, including 1) arginase‐1 (Arg1) and inducible nitric oxide synthase (iNOS)‐driven depletion of essential T‐cell metabolites, 2) secretion of immunosuppressive cytokines such as IL‐10 and TGF‐β, and 3) excessive production of reactive oxygen species (ROS).^[^ ^11^ ^]^ Their recruitment to the tumor microenvironment is tightly regulated by a complex chemokine network.^[^ ^12^, ^13^ ^]^ Accumulating evidence suggests that MDSCs may also drive resistance to immune checkpoint inhibitors (ICIs), whereas blockade of MDSC‐mediated immunosuppression can increase ICI efficacy in patients with advanced colorectal cancer (CRC).^[^ ^14^ ^]^ Given their central role in maintaining an immunosuppressive tumor milieu, therapeutic targeting of MDSCs through depletion or functional inhibition has emerged as a promising strategy to potentiate cancer immunotherapy.

In this study, we revealed the critical link between tumor‐intrinsic KDM6B and MDSC accumulation in the CRC microenvironment: KDM6B deficiency exacerbated tumor progression and increased MDSCs accumulation, as validated in KDM6B transgenic mice and syngeneic mouse models. Through integrated RNA‐seq, CUT&Tag sequencing, and cytokine profiling, we elucidated the mechanistic role of the KDM6B–SLC10A2 axis in recruiting immunosuppressive MDSCs to promote tumor growth. Clinically, we demonstrated that low KDM6B/SLC10A2 expression is correlated with MDSC infiltration and poor prognosis in human CRC specimens. Finally, we demonstrated that targeting KDM6B in CRC may increase the efficacy of ICB therapy, providing a promising avenue for improving patient outcomes. These findings position KDM6B as both a prognostic biomarker and a therapeutic target for overcoming immunosuppression in CRC.

## 2 Results

### 2.1 KDM6B Deficiency Exacerbates CRC Progression and Promotes an Immunosuppressive Microenvironment

**Figure 1:**
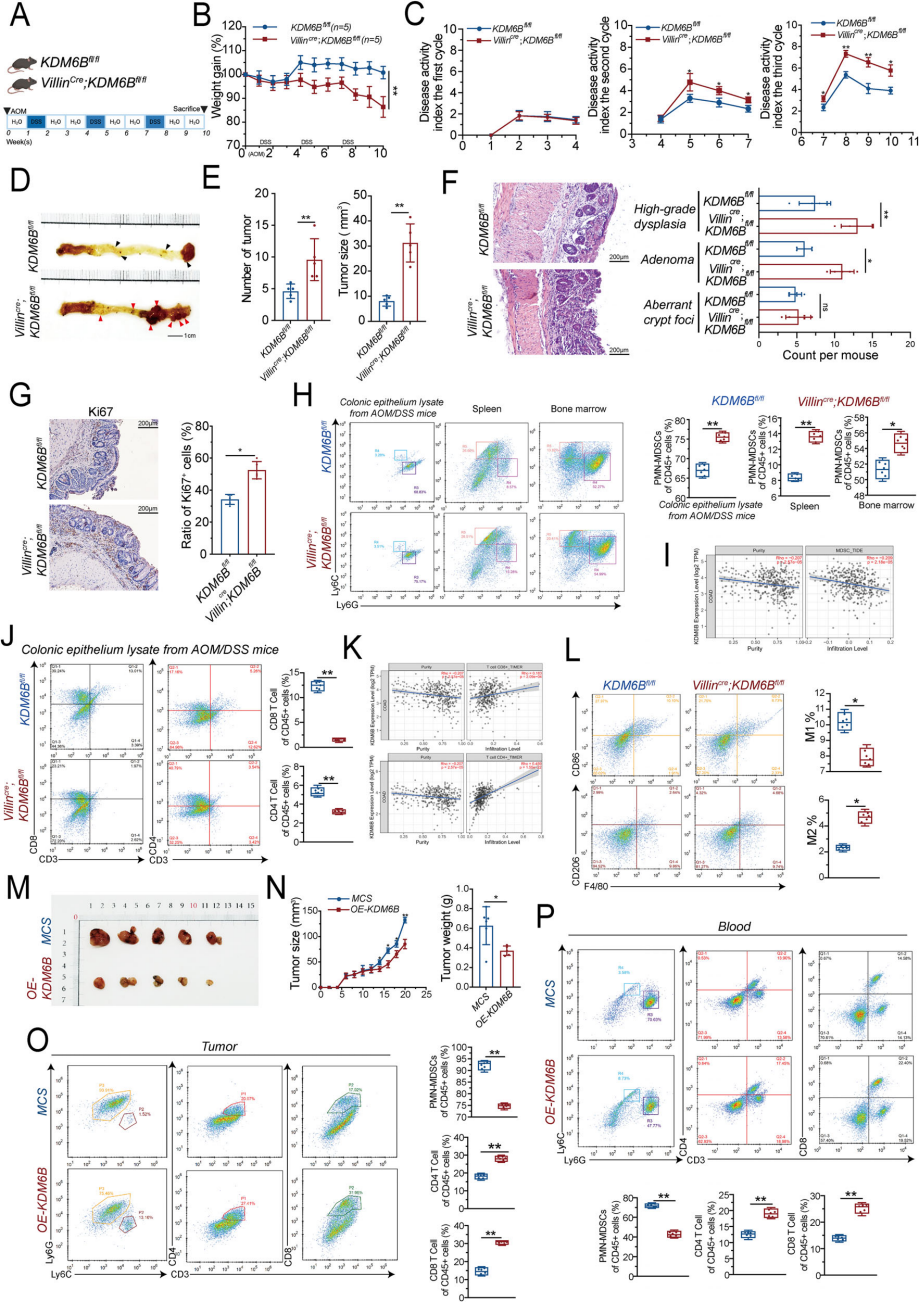
KDM6B deficiency exacerbates CRC progression and promotes an immunosuppressive microenvironment. A) Schematic of the AOM/DSS‐induced CRC model in the Villin^Cre^; KDM6B^fl/fl^ group and KDM6B^fl/fl^ group; each genotype was represented by five biological replicates. B) Longitudinal body weight changes. C) Disease activity index (DAI) scores (0–4 scale) during three DSS cycles. D) Macroscopic view of colon tumors at the endpoint. The pathological area and manifestation of tumors in the colon are indicated by arrows. E) Quantification of tumor number (left) and diameter (right). F) H&E staining of colon tissues. Grading of dysplastic lesions (aberrant crypt foci, adenoma, and high‐grade dysplasia). G) Left: Representative immunohistochemical staining of Ki67 in colon crypts. The black arrows indicate Ki67^+^ proliferating cells. Right: Quantification of Ki67^+^ cells per crypt. H) Flow cytometry gating strategy for PMN‐MDSCs (CD11b^+^Ly6G^+^) from the colon epithelium, spleen, and bone marrow. I) Quantification of PMN‐MDSC frequencies and correlation between KDM6B expression and MDSC infiltration in human CRC using the TIMER2.0 database. (J) Flow cytometry of CD4^+^ and CD8^+^ T cells in the colon epithelium. K) Correlations between KDM6B expression and CD4/CD8 T‐cell markers in human CRC according to the TIMER2.0 database. L) Flow cytometry analysis of M1 (CD86^+^) and M2 (CD206^+^) macrophages in the colon epithelium. M) Representative subcutaneous tumors from the MC38‐vector and MC38‐KDM6B‐OE groups. N) Tumor growth curves and endpoint tumor weights. O) Flow cytometry of tumor‐infiltrating PMN‐MDSCs and CD4^+^/CD8^+^ T cells. P) Flow cytometry of peripheral blood PMN‐MDSCs and CD4^+^/CD8^+^ T cells. Scale bars: 200 µm. Data are pooled from five independent mice in (B–K) and presented as the means ± SEM. Statistical significance was determined by an unpaired, two‐tailed Student's *t* test. ^*^ *p* < 0.05, ^**^ *p* < 0.01. (A) was created with bioRender.com, with permission.

To elucidate the role of KDM6B in colorectal cancer (CRC) progression and its effect on the immunosuppressive microenvironment, we generated intestinal epithelium–specific KDM6B knockout mice (Villin^Cre^; KDM6B^fl/fl^) and induced CRC using the azoxymethane/dextran sulfate sodium salt (AOM/DSS) model (**Figure** **1**A). Longitudinal assessment revealed that loss of KDM6B resulted in significant weight loss (Figure 1B) and increased disease activity index (DAI) scores (Figure 1C) beginning at week 4 post‐induction, which indicated accelerated disease progression. KDM6B deficiency markedly promoted colon tumor formation, leading to increased tumor number and size (Figure 1D,E). Histopathological examination revealed a pronounced shift toward high‐grade dysplasia in KDM6B‐deficient mice (Figure 1F). Consistent with these findings, immunohistochemical staining for Ki67 revealed increased epithelial proliferation in the KDM6B‐deficient colon (Figure 1G).

We further determined whether KDM6B modulates the immune microenvironment of CRC. Flow cytometry analysis revealed substantial expansion of MDSCs in KDM6B‐deficient mice, including both PMN‐MDSCs (CD11b⁺Ly6G⁺Ly6C^−^) and M‐MDSCs (CD11b⁺Ly6G^−^Ly6C⁺) in the colonic epithelium, spleen, and bone marrow (Figure 1H; Extended Figure 1A). The PMN‐MDSCs exhibited the most pronounced increase, while M‐MDSCs showed a moderate but consistent increase. This systemic myeloid expansion was accompanied by a marked reduction in both CD4⁺ and CD8⁺ T‐cell populations (Figure 1J), which is consistent with an immunosuppressive shift. Bioinformatic correlation analysis further revealed a significant negative correlation between KDM6B expression and MDSC infiltration in human CRC datasets (Figure 1I). Moreover, KDM6B deficiency promoted macrophage polarization toward the M2 phenotype (CD11b⁺F4/80⁺CD206⁺) while suppressing M1 macrophages (CD11b⁺F4/80⁺CD86⁺) (Figure 1L), reinforcing the establishment of an immunosuppressive tumor microenvironment.

To exclude the possibility that epithelial KDM6B deletion intrinsically disrupts intestinal homeostasis, we analyzed untreated Villin^Cre^; KDM6B^fl/fl^ mice. Histological and immunohistochemical analyses confirmed normal crypt–villus architecture, preserved expression of tight junction markers (Occludin and ZO‐1), no significant changes in intestinal stem cell (ISC)‐mediated renewal (CD44), comparable Ki67⁺ proliferation rates, and an absence of spontaneous immune infiltration (Extended Figure 1C–G). These findings indicate that epithelial KDM6B loss does not compromise intestinal renewal, barrier integrity, or immune quiescence under physiological conditions.

To extend these findings beyond inflammation‐driven carcinogenesis, we used an orthotopic colorectal tumor implantation model in which MC38 cells were orthotopically injected into the rectal submucosa of C57BL/6 mice. Consistent with the results of the AOM/DSS models, compared with the negative control (NC), KDM6B knockdown (sh‐KDM6B) accelerated orthotopic tumor growth, whereas KDM6B overexpression (OE‐KDM6B) significantly suppressed tumor progression (Figure S2A–C, Supporting Information). Immunohistochemical analysis revealed that KDM6B knockdown (sh‐KDM6B) markedly increased Gr1⁺ MDSC infiltration, whereas KDM6B overexpression (OE‐KDM6B) reduced Gr1⁺ MDSC infiltration (Figure S2D, Supporting Information). To further validate the regulatory role of KDM6B in CRC progression and immune modulation, we established subcutaneous tumor models using MC38 cells overexpressing KDM6B (OE‐KDM6B) or vector control (MCS). KDM6B overexpression significantly attenuated tumor growth (Figure 1M,N) and selectively reduced PMN‐MDSC infiltration, whereas the M‐MDSC frequency remained largely unaffected (Figure 1O,P, Extended Figure 1B). This selective suppression of PMN‐MDSCs was accompanied by a notable increase in both CD4⁺ and CD8⁺ T‐cell infiltration in tumor tissues and peripheral blood, suggesting that KDM6B may primarily constrain the granulocytic subset of MDSCs to restore antitumor immunity.

Collectively, these data demonstrate that epithelial KDM6B loss may promote colorectal tumorigenesis by remodeling the tumor immune microenvironment by driving the expansion of both MDSC subsets, particularly PMN‐MDSCs, while suppressing antitumor T‐cell responses. The consistency of these effects across AOM/DSS, orthotopic, and subcutaneous models underscores the robustness of KDM6B as a key determinant of CRC progression.

### 2.2 RNA‐Seq Reveals KDM6B‐Mediated Chemokine Regulation Through TNF/AP1 Signaling

**Figure 2:**
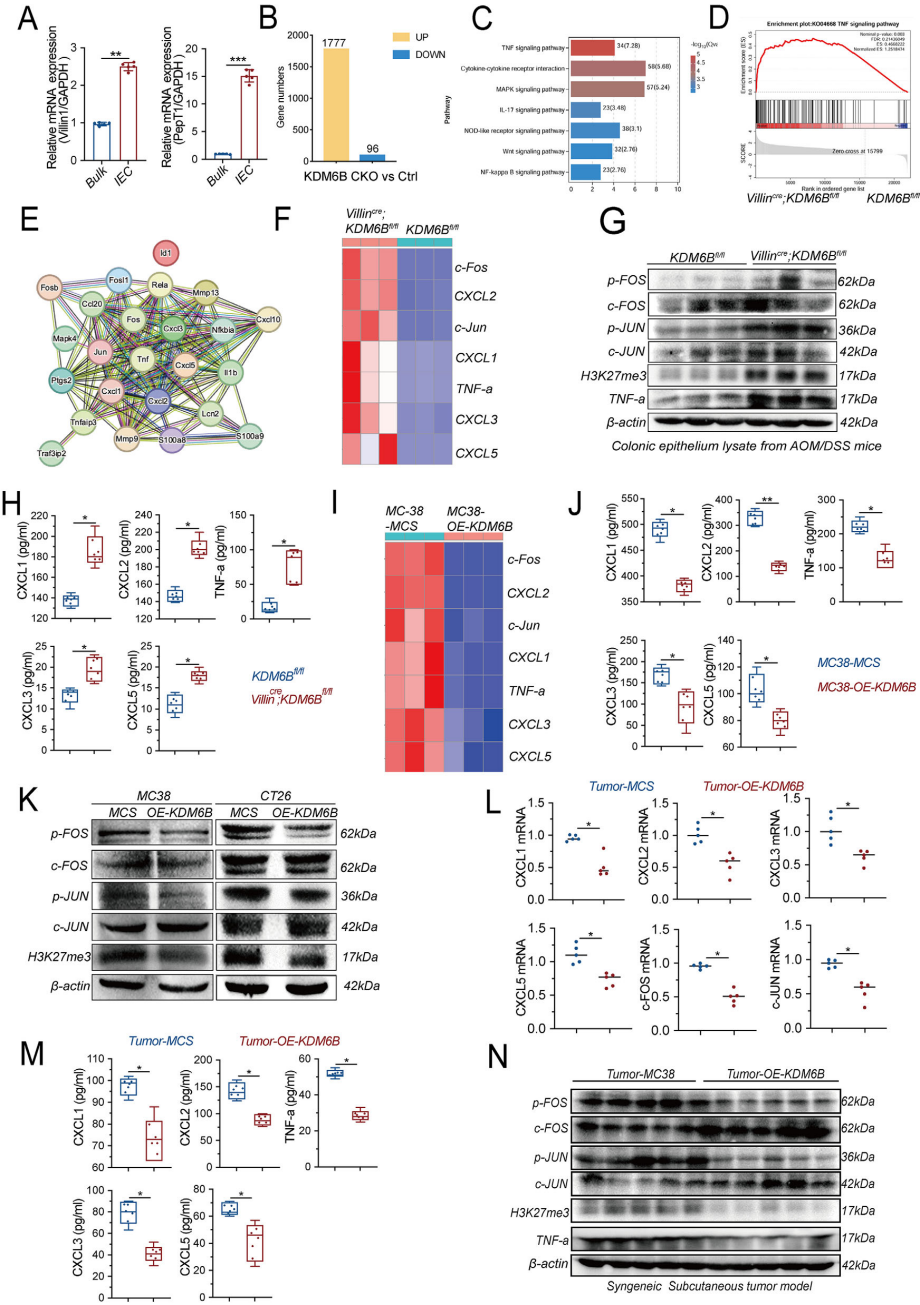
RNA‐seq reveals KDM6B‐mediated chemokine regulation through TNF/AP1 signaling. A) Quantitative PCR (qPCR) analysis of the intestinal epithelial cell (IEC) markers Villin1 and PepT1 in purified IECs isolated from Villin^Cre^; KDM6B^fl/fl^ mice and KDM6B^fl/fl^ littermate controls after 10 weeks of AOM/DSS treatment. n = 5 mice per group. B) Volcano plot of differentially expressed genes (DEGs) identified by RNA‐seq in IECs from Villin^Cre;^ KDM6B^fl/fl^ mice (KDM6B CKO) compared with KDM6B^fl/fl^ controls (Ctrl) after 10 weeks of AOM/DSS treatment (n = 5 mice per group). Criteria: |log_2_FC| > 1 and adjusted p value < 0.05. A total of 1777 genes were upregulated and 96 genes were downregulated in KDM6B‐deficient IECs, and each genotype was represented by five biological replicates. C) KEGG pathway enrichment analysis of the regulated DEGs. The top 7 significantly enriched pathways were shown. D) Gene set enrichment analysis (GSEA) plots showing that compared with that in the control group, TNF‐α signaling was significantly enriched in Villin^Cre^; KDM6B^fl/fl^ mice (normalized enrichment score [NES]; false discovery rate [FDR], as shown in the figure). E) STRING network analysis of the top 24 genes in the TNF‐α pathway using STRING website (https://cn.string‐db.org/). (F–G) TNF‐α pathway activation in KDM6B‐deficient IECs. F) qPCR heatmap of TNF‐α‐related genes in AOM/DSS‐induced colon tissues (Villin^Cre^; KDM6B^fl/fl^ vs KDM6B^fl/fl^). G) Western blot analysis of the protein levels of p‐FOS, c‐FOS, p‐JUN, c‐JUN, H3K27me3, TNF‐a, and β ‐actin. H) Serum chemokine levels of CXCL (1, 2, 3, and 5) and TNF‐α determined by ELISA (Villin^Cre^; KDM6B^fl/fl^ vs KDM6B^fl/fl^). (I–K) TNF‐α pathway modulation in MC38/CT26 cells: I) Heatmap analysis of the mRNA expression of TNF‐α pathway‐related genes in MC38‐vector (MC38‐MCS) vs MC38‐OE‐KDM6B cells as determined by qPCR (n = 3). J) Levels of CXCL (1, 2, 3, and 5) in cell supernatants from MC38‐vector (MC38‐MCS) and MC38‐overexpressing KDM6B cells (MC38‐OE‐KDM6B). K) Western blot analysis of p‐FOS, c‐FFOS, p‐JUN, c‐JUN, H3K27me3, and β‐actin expression in MC38‐ and CT26‐ vector control (MCS) or KDM6B‐overexpressing (OE‐KDM6B) cells. L–N) In vivo validation: (L) qPCR analysis of the mRNA expression of the chemokines CXCL (1, 2, 3, and 5) and c‐Fos and c‐Jun in subcutaneous tumors inoculated with MC38‐MCS and MC38‐OE‐KDM6B cells. M) Serum levels of the chemokines CXCL (1, 2, 3, and 5) and TNF‐α were measured by ELISA in tumor‐bearing mice inoculated with MC38‐MCS or MC38‐OE‐KDM6B cells. N) Western blot analysis of p‐FOS, c‐FOS, p‐JUN, c‐JUN, H3K27me3, TNF‐α and β‐actin expression in tumor tissues from tumor‐bearing mice inoculated with MC38‐MCS or MC38‐OE‐KDM6B cells. The sample sizes for (H, J, L, M) were n = 5. Statistical analysis was performed using independent *t* tests for (A, H, J, L, M). ^*^ *p* < 0.05, ^**^ *p* < 0.01, ^***^ *p* < 0.001.

 To elucidate the molecular mechanisms through which epithelial KDM6B shapes the tumor immune microenvironment, intestinal epithelial cells (IECs) were isolated from AOM/DSS‐induced Villin^Cre^; KDM6B^fl/fl^ and control (KDM6B^fl/fl^) mice (**Figure** **2**A) and subjected to transcriptomic profiling. Differential expression analysis revealed 1873 genes whose expression was altered in KDM6B‐deficient IECs, including 1777 upregulated and 96 downregulated genes (Figure 2B). Gene set enrichment analysis (GSEA) revealed significant enrichment of the TNF signaling pathway (Figure 2C,D), while network analysis of TNF‐associated genes revealed a core regulatory module centered on TNF that incorporated Fos, Jun, and C‐X‐C motif chemokin ligand (CXCL) chemokines (CXCL1, 2, 3, and 5) (Figure 2E).

We subsequently conducted experimental verification. qPCR validation confirmed increased mRNA levels of c‐FOS, c‐JUN, and CXCL (1, 2, 3, and 5) in KDM6B‐deficient IECs (Figure 2F), accompanied by concordant increases in their phosphorylated forms (p‐Fos and p‐Jun) and TNF‐α protein levels, as well as increased H3K27me3 enrichment (Figure 2G). Consistently, serum concentrations of CXCL (1, 2, 3, and 5) and TNF‐α were significantly greater in Villin^Cre^; KDM6B^fl/fl^ mice than in control mice (Figure 2H), suggesting that loss of epithelial KDM6B amplifies proinflammatory cytokine signaling.

Conversely, KDM6B overexpression in MC38 cells suppressed the expression of c‐FOS, c‐JUN, CXCL (1, 2, 3, and 5), and TNF‐α at both the mRNA (Figure 2I) and protein levels (p‐FOS, p‐JUN, TNF‐α) (Figure 2K) and markedly reduced their secretion (CXCL (1, 2, 3, and 5) and TNF‐α) (Figure 2J). Similar inhibitory effects were observed in subcutaneous tumor models derived from OE‐KDM6B‐MC38 cells, which exhibited decreased transcription of CXCL (1, 2, 3, and 5), c‐FOS, and c‐JUN (Figure 2L), reduced levels of circulating CXCL chemokines and TNF‐α (Figure 2M), and decreased protein levels of p‐FOS, p‐JUN, TNF‐α, and H3K27me3 in tumor tissues (Figure 2N).

c‐FOS and c‐JUN are key components of the transcription factor complex AP‐1, which transcriptionally regulates downstream gene expression. To investigate the transcriptional regulatory role of AP‐1 on CXCL (1, 2, 3, and 5), a ChIP‒qPCR assay was performed. The results confirmed the binding of the AP‐1 component c‐FOS to the promoter regions of CXCL1, CXCL2, CXCL3, and CXCL5, with significantly increased enrichment following KDM6B knockdown (Extended Figure 2). These results provide direct mechanistic evidence that KDM6B loss increases AP‐1–mediated transcriptional activation of CXCL chemokine genes.

Collectively, these findings demonstrate that epithelial KDM6B depletion activates AP‐1 signaling, thereby promoting CXCL chemokine expression and fostering a proinflammatory and immunosuppressive tumor microenvironment.

### 2.3 KDM6B Deficiency Promotes MDSCs‐Mediated Immunosuppression Via the AP‐1/CXCL–CXCR2 Axis

**Figure 3:**
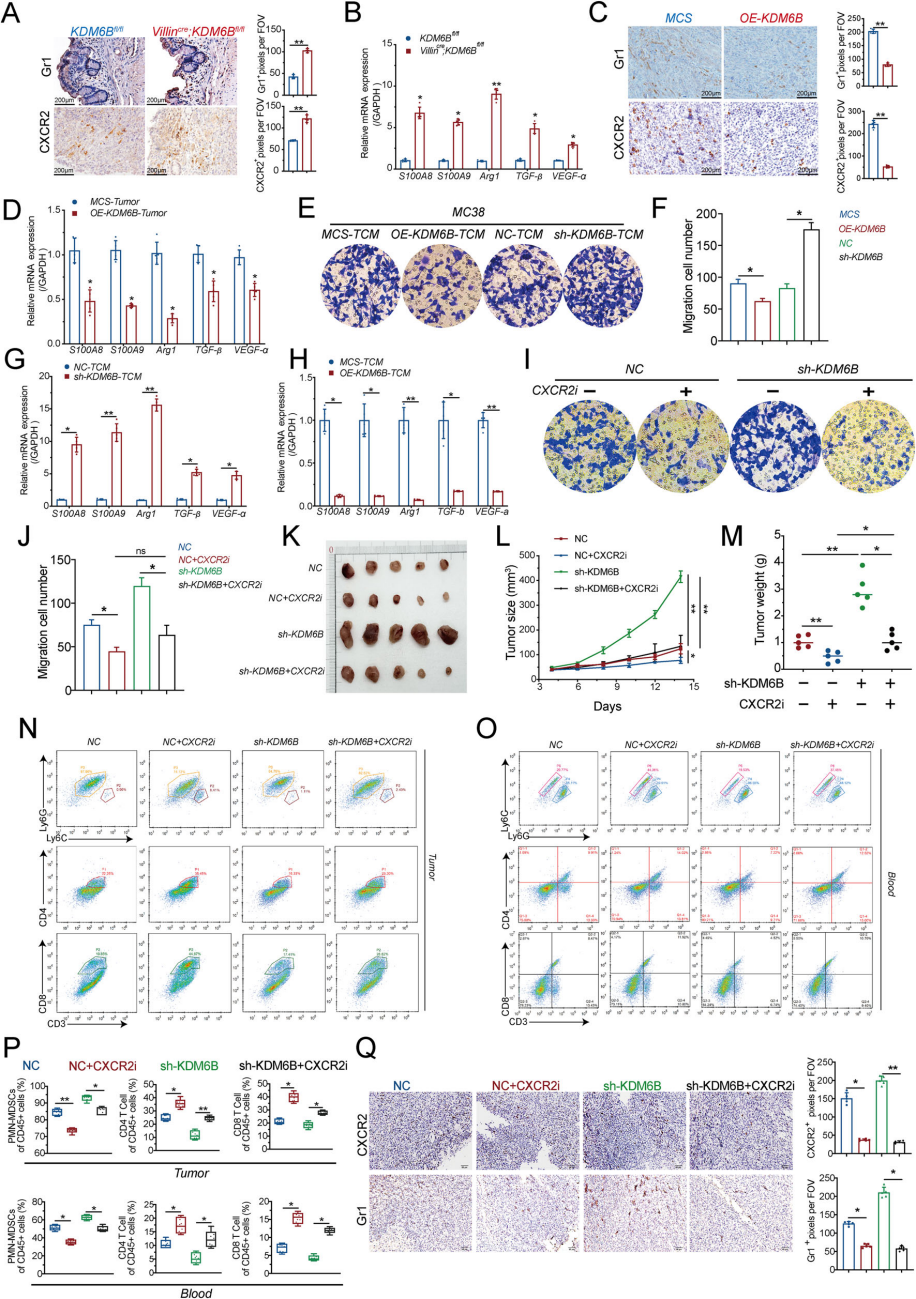
KDM6B deficiency promotes MDSCs‐mediated immunosuppression via the AP‐1/CXCL–CXCR2 axis. A) Immunohistochemical (IHC) staining of Gr1 and CXCR2 in colon tissues from Villin^Cre^; KDM6B^fl/fl^ mice and KDM6B^fl/fl^ littermate controls after 10 weeks of AOM/DSS treatment. Quantification of positive pixels per field. B) qPCR analysis of the expression of MDSC markers (S100A8, S100A9, Arg1, TGF‐β, and VEGF‐a) in the intestinal epithelium of Villin^Cre^; KDM6B^fl/fl^ mice and KDM6B^fl/fl^ mice. C,D) MC38 murine colon cancer cells stably overexpressing KDM6B (OE‐KDM6B) or empty vector control (MCS) were subcutaneously injected into C57BL/6 mice (n = 5 per group). (C) Immunohistochemical (IHC) staining of Gr1 and CXCR2 in subcutaneous tumors derived from MC38‐MCS and MC38‐OE‐KDM6B cells at the endpoint (14 days post‐injection). Representative images are shown. Scale bars: 200 µm. D) qPCR of the expression of MDSCs markers (S100A8, S100A9, Arg1, TGF‐β, and VEGF‐a) in subcutaneous tumors derived from MC38‐MCS and MC38‐OE‐KDM6B cells at the endpoint. E) Transwell migration assays were performed to evaluate the ability of tumor‐conditioned media (TCM) from MC38 cells, including the MC38‐vector (MCS), MC38‐OE‐KDM6B (OE‐KDM6B), MC38‐shNC (NC), and MC38‐shKDM6B (sh‐KDM6B) groups, to recruit MDSCs. Representative images are shown. Each group had three biological replicates. F) Quantification of migrated MDSCs. G,H) qPCR of the expression of MDSC markers (S100A8, S100A9, Arg1, TGF‐β, and VEGF‐a) after TCM treatment. I,J) CXCR2 blockade experiments: (I) A CXCR2 inhibitor (SB265610, 10 µM) was added into tumor‐conditioned media (TCM) from MC38‐NC cells or MC38‐sh‐KDM6B cells, and Transwell assays were performed to evaluate the migration capacity of MDSCs. Representative images are shown. Each group had three biological replicates. (J) The statistical results of the migration cell count. K–M) In vivo CXCR2 inhibition: (K) Subcutaneous tumors were generated in C57BL/6 mice by injection of MC38 cells with stable KDM6B knockdown (sh‐KDM6B) or negative control (NC). Mice were treated daily with the CXCR2 inhibitor SB265610 (2 mg kg^−1^ day^−1^, i.p.) or vehicle control for 15 days (n = 5). (L) Tumor growth curves. (M) Endpoint tumor weights. (N) Flow cytometry of tumor‐infiltrating PMN‐MDSCs (CD11b^+^Ly6G^+^Ly6C^−^), CD4^+^ T cells (CD3^+^CD4^+^) and CD8^+^ T cells (CD3^+^CD8^+^). (O) Flow cytometry of peripheral blood from the four experimental groups (NC, NC+CXCR2i, sh‐KDM6B, and sh‐KDM6B+CXCR2i) was performed at the endpoint (Day 15). Gating strategy for PMN‐MDSCs (CD11b⁺Ly6G⁺Ly6C^−^), CD4^+^ T cells (CD3^+^CD4^+^) and CD8^+^ T cells (CD3^+^CD8^+^). (P) Quantification of MDSCs and T cells in tumors and peripheral blood. (Q) Immunohistochemical (IHC) analysis of CXCR2 and Gr1⁺ cell infiltration in subcutaneous tumors. The sample size for each group was n = 5. The middle line in the box plots represents the median, whereas the whiskers denote the minimum‐to‐maximum range of the data distribution. The error bars show the means ± SEM. Statistical significance was determined by an unpaired, two‐tailed Student's *t* test. ^*^ *p* < 0.05, ^**^ *p* < 0.01.

Given that CXCL (1, 2, 3, and 5) chemokines recruit MDSCs via CXCR2, we hypothesized that KDM6B deletion increases MDSCs infiltration through activation of the AP‐1/CXCL–CXCR2 axis, thereby fostering an immunosuppressive tumor microenvironment and promoting colorectal tumor progression. To test this hypothesis, we first evaluated the effect of KDM6B on MDSC infiltration. Immunohistochemical staining analysis revealed that KDM6B deficiency markedly increased Gr1⁺ MDSC infiltration and upregulated CXCR2 expression in colonic tumors (**Figure** **3**A). These findings were corroborated by CD11b/Gr1 costaining, providing more specific evidence of MDSC accumulation (Extended Figure 3A). Consistently, the results of the qPCR analysis revealed increased expression of MDSC‐associated immunosuppressive mediators, including S100A8, S100A9, Arg1, TGF‐β, and VEGF‐a (Figure 3B). Conversely, KDM6B overexpression in subcutaneous tumor models significantly reduced Gr1⁺ and CXCR2⁺ cell infiltration and suppressed the transcription of these immunoregulatory molecules (Figure 3C,D).

We next assessed the role of KDM6B in MDSC recruitment and function in vitro. The results showed that conditioned medium from KDM6B‐overexpressing MC38 cells markedly inhibited MDSC migration in Transwell assays, whereas medium derived from KDM6B‐silenced cells increased migration (Figure 3E,F), which is consistent with the observed downregulation or upregulation of the expression of CXCL chemokines (Figure 3G,H). Moreover, MDSCs preconditioned with shKDM6B‐derived medium exhibited stronger suppression of CD8⁺ IFN‐γ⁺ T‐cell activation (Figure S3A–D, Supporting Information), confirming that the loss of KDM6B increases both MDSC recruitment and their immunosuppressive functionality.

To clarify which specific CXCL ligand is functionally dominant, we performed Transwell migration assays. Conditioned media from NC‐MC38 or sh‐KDM6B‐MC38 cells were placed in the lower chamber, with or without neutralizing antibodies against CXCL1, CXCL2, CXCL3, or CXCL5. MDSCs were placed in the upper chamber to assess migration. The results revealed CXCL1 and CXCL2 as the predominant ligands driving MDSC chemotaxis downstream of KDM6B loss, whereas CXCL3 and CXCL5 played minor roles (Figure S4, Supporting Information).

To verify the role of CXCR2‐mediated MDSCs chemotaxis, MDSCs were exposed to conditioned medium from KDM6B‐silenced MC38 cells in the presence or absence of a selective CXCR2 inhibitor. Pharmacologic blockade of CXCR2 expression significantly attenuated the MDSCs migration induced by KDM6B knockdown (Figure 3I,J). In vivo, CXCR2 inhibition similarly mitigated the tumor‐promoting effects of KDM6B loss, resulting in markedly reduced tumor growth and weight in subcutaneous models (Figure 3K–M). Flow cytometry revealed that KDM6B knockdown primarily expanded the PMN‐MDSCs (CD11b⁺Ly6G⁺Ly6C^−^) subset while depleting CD4⁺ and CD8⁺ T cells, and these changes were effectively reversed by CXCR2 inhibition (Figure 3N–P). A compensatory increase in M‐MDSCs (CD11b⁺Ly6G^−^Ly6C⁺) was also observed following CXCR2 blockade (Extended Figure 3B).

These results indicated that KDM6B deletion increased MDSC infiltration through activation of the AP‐1/CXCL–CXCR2 axis. To establish a causal link between MDSCs and KDM6B‐mediated tumorigenesis, we performed anti‐Gr1 antibody‐mediated MDSC depletion in tumor‐bearing mice. The results showed that depletion of MDSCs markedly abrogated the increased tumor growth observed in KDM6B‐knockdown tumors compared with that in isotype controls (Figure S5A–C, Supporting Information), confirming that the protumor effects of KDM6B loss are largely MDSCs dependent.

Collectively, these results provide convergent mechanistic evidence that KDM6B deficiency drives MDSCs accumulation and activation predominantly through the CXCL1/2–CXCR2 signaling axis, thereby establishing a profoundly immunosuppressive microenvironment that promotes CRC progression.

### 2.4 CUT&Tag and RNA‐Seq Analysis Reveal that the H3K27me3 Level of SLC10A2 is Regulated by KDM6B in CRC

**Figure 4:**
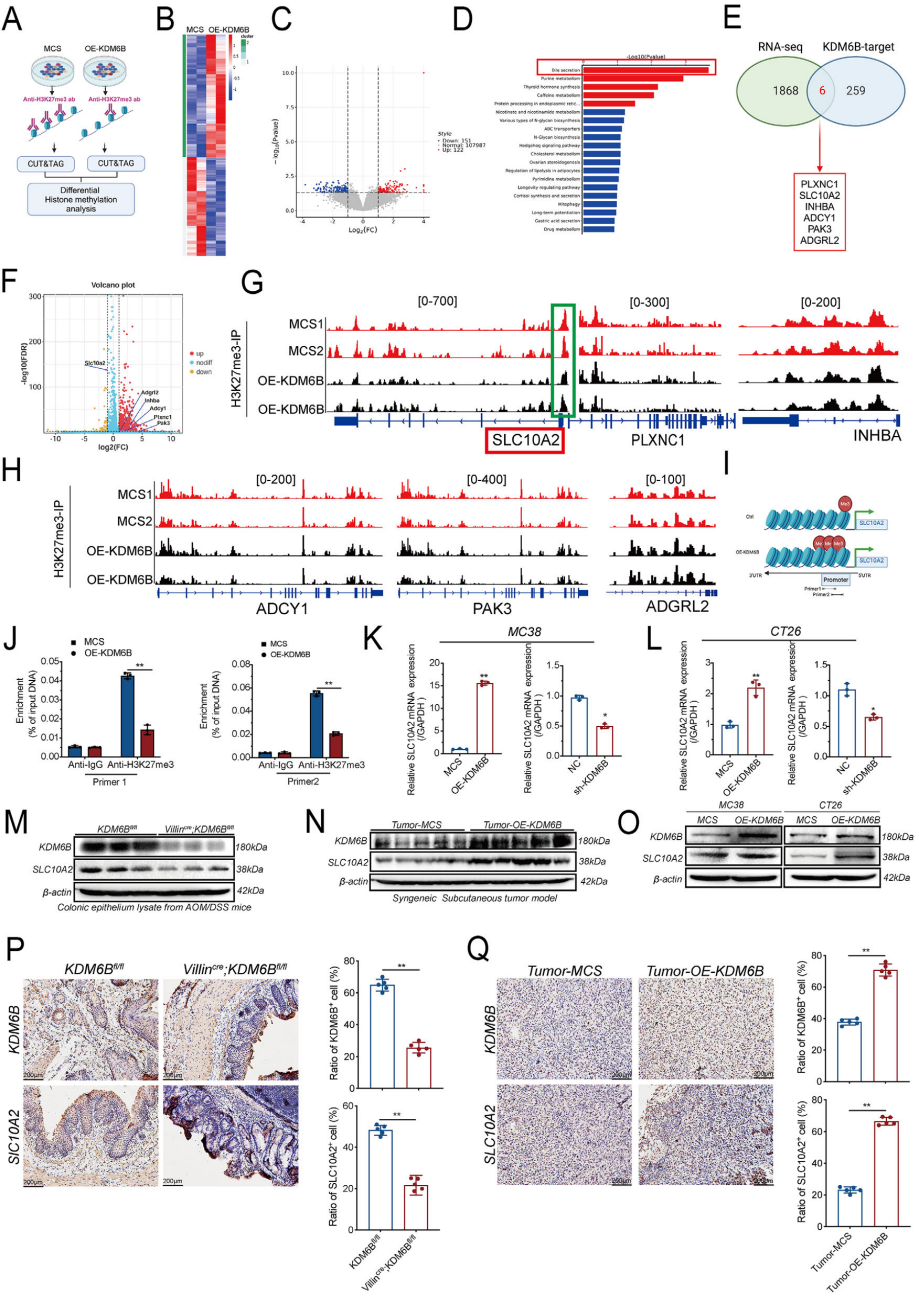
CUT&Tag and RNA‐seq analyses reveal KDM6B‐mediated H3K27me3 methylation of SLC10A2 in CRC. A) Schematic of the CUT&Tag sequencing workflow comparing HCT116‐vector control (MCS) and HCT116‐OE‐KDM6B cells (created with bioRender.com, with permission.). Each genotype was represented by two biological replicates. B) Heatmap of differential chromatin peaks in HCT116‐vector (MCS) and HCT116‐OE‐KDM6B cells. C) Volcano plot showing 151 downregulated and 122 upregulated peaks in OE‐KDM6B cells compared with those in MCS cells. D) KEGG pathway enrichment analysis of differential peaks identified by CUT&Tag sequencing. E) Integrated analysis of RNA‐seq and CUT&Tag data identifying 6 coregulated genes (PLXNC1, SLC10A2, INHBA, ADCY1, PAK3, and ADGRL2). F) RNA‐seq volcano plot confirming SLC10A2 as the most significantly downregulated gene upon KDM6B deficiency. G) IGV visualization of H3K27me3 CUT&Tag‐seq profiles at the PLXNC1, SLC10A2, and INHBA gene loci. H) IGV visualization of H3K27me3 CUT&Tag‐seq profiles at the ADCY1, PAK3, and ADGRL2 gene loci. I) ChIP‒qPCR analysis of H3K27me3 enrichment at the SLC10A2 promoter in control and KDM6B‐overexpressing MC38 cells. J) Reduced H3K27me3 enrichment at the SLC10A2 gene promoter in OE‐KDM6B cells. The sample size for each group was n = 3. K) qPCR analysis of SLC10A2 mRNA expression in MC38 cells with KDM6B overexpression or knockdown. The sample size for each group was n = 3. L) qPCR analysis of SLC10A2 mRNA expression in CT26 cells with KDM6B overexpression or knockdown. The sample size for each group was n = 3. M) Western blot analysis of KDM6B and SLC10A2 expression in intestinal tumor tissues from Villin^Cre^; KDM6B^fl/fl^ mice and KDM6B^fl/fl^ mice (n = 3), N) Western blot analysis of subcutaneous tumors derived from MC38‐vector control (MCS) and MC38‐OE‐KDM6B cells (n = 5). O) Western blot analysis of KDM6B and SLC10A2 expression in MC38/CT26‐OE‐KDM6B or vector control (MCS) cells. P) IHC analysis of KDM6B and SLC10A2 in colon tissues from Villin^Cre^; KDM6B^fl/fl^ mice and KDM6B^fl/fl^ mice. Representative images and percentages of KDM6B^+^ and SLC10A2^+^ cells were shown, and unpaired two‑sided *t* test was used; n = 5 per group. Q) IHC analysis of KDM6B and SLC10A2 in subcutaneous tumors derived from MC38‐vector control (MCS) and MC38‐OE‐KDM6B cells. Representative images and percentages of KDM6B^+^ and SLC10A2^+^ cells were shown. And unpaired two‐sided *t* test was used; n = 5 per group. ^*^ *p* < 0.05, ^**^ *p* < 0.01. Statistical analysis was performed using a dependent *t* test for (J–L). ^*^ *p* < 0.05, ^**^ *p* < 0.01. All error bars represent the means ± SEM. Images for (A, I) were created with bioRender.com, with permission.

To delineate the transcriptional targets of KDM6B, we performed an integrated analysis of the RNA‐seq and CUT&Tag datasets. CUT&Tag profiling of HCT116 cells overexpressing KDM6B (OE‐KDM6B) versus vector control cells (MCS) using an H3K27me3 antibody revealed distinct alterations in the histone methylation landscape (**Figure** **4**A). Differential peak analysis revealed widespread redistribution of H3K27me3 marks, and KEGG enrichment of peak‐proximal genes highlighted bile secretion as the most significantly affected pathway (Figure 4B–D), suggesting an unexpected link between KDM6B‐mediated epigenetic remodeling and bile acid metabolism in colorectal cancer.

Intersection of RNA‐seq data (1868 differentially expressed genes) with CUT&Tag peaks (259 KDM6B‐targeted loci) yielded six overlapping candidate genes (Figure 4E). Among these genes, SLC10A2 emerged as the most compelling target on the basis of its strong downregulation according to RNA‐seq (Figure 4F), marked loss of H3K27me3 at its promoter in HCT116‐OE‐KDM6B cells (Figure 4G,H), and functional relevance to the bile secretion pathway. ChIP‒qPCR analysis further confirmed reduced H3K27me3 occupancy at two SLC10A2 promoter regions in MC38‐OE‐KDM6B cells (Figure 4I,J), whereas KDM6B knockdown significantly increased H3K27me3 enrichment (Figure S6A, Supporting Information). Similarly, KDM6B overexpression in MDSCs reduced H3K27me3 levels on the SLC10A2 promoter (Figure S6B, Supporting Information), establishing a direct epigenetic regulatory relationship between KDM6B and SLC10A2. Notably, this mechanism was conserved in murine CT26 cells, where KDM6B overexpression also decreased H3K27me3 enrichment at the SLC10A2 promoter (Figure S6C, Supporting Information), confirming the cross‐species conservation of this regulatory axis.

Functional validation across multiple systems revealed a strong positive correlation between KDM6B and SLC10A2 expression at both the mRNA (Figure 4K,L) and protein levels (Figure 4M–O). Immunohistochemical and immunofluorescence staining analyses provided consistent evidence across intestinal tissues, subcutaneous tumors, and CRC cell lines, with quantitative image analysis confirming significantly increased KDM6B and SLC10A2 signaling in the OE‐KDM6B groups (Figure 4P,Q, Extended A).

Collectively, these findings establish SLC10A2 as a bona fide epigenetic target of KDM6B that is regulated by H3K27me3 demethylation at its promoter. This regulatory mechanism highlights a novel epigenetic link between KDM6B activity and its downstream target SLC10A2 in colorectal cancer, providing critical insights into the KDM6B‐mediated transcriptional regulatory network in this malignancy.

### 2.5 SLC10A2 Reverses KDM6B Deficiency‐Induced AP‐1/CXCL–MDSCs Immunosuppression

**Figure 5:**
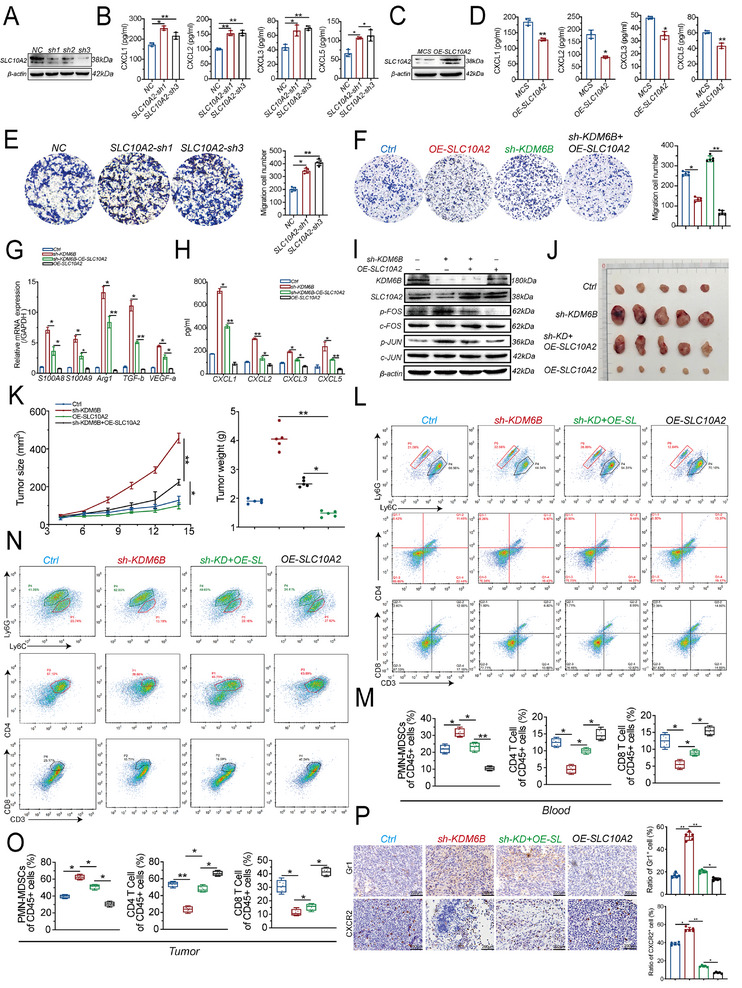
SLC10A2 reverses KDM6B deficiency‐induced AP1/CXCL‐MDSC immunosuppression. A) Western blot analysis of SLC10A2 knockdown efficiency in MC38 cells using three independent shRNAs (sh1, sh2, and sh3). β‐actin served as a loading control. B) ELISA quantification of CXCL (1, 2, 3, and 5) secretion in conditioned media from MC38‐negative control (NC), SLC10A2‐sh1 and SLC10A2‐sh3 cells. C) Western blot confirmation of SLC10A2 overexpression in MC38 cells. β‐actin was used as a loading control. D) ELISA analysis of CXCL (1, 2, 3, and 5) secretion in SLC10A2‐overexpressing MC38 cells. E) Transwell migration assay showing MDSC recruitment by conditioned media from MC38‐negative control (NC), SLC10A2‐sh1 and SLC10A2‐sh3 cells. The right panel shows the statistical results. Each group had three biological replicates. F) Comparison of MDSC migration across four groups of MC38 cells: control, OE‐SLC10A2, sh‐KDM6B, and sh‐KDM6B+OE‐SLC10A2. The right panel shows the statistical results. Each group had three biological replicates. G) qPCR analysis of the expression of MDSC markers (S100A8, S100A9, Arg1, TGF‐β, and VEGF‐a) following stimulation with tumor supernatant. H) Chemokine secretion profiles across the four treatment groups determined by ELISA. I) Western blot analysis of KDM6B, SLC10A2, p‐FOS, c‐FOS, p‐JUN, c‐JUN and β‐actin expression. J) MC38 cells were subcutaneously injected into C57BL/6 mice to establish syngeneic transplant tumors. The experimental groups were Ctrl, sh‐KDM6B, sh‐KDM6B+OE‐SLC10A2, and OE‐SLC10A2. The mice (n = 5 per group) were sacrificed on Day 15 post‐injection, and the tumors were harvested for further analysis. K) Tumor growth kinetics (left) and endpoint weights (right) for the four treatment groups. L) Flow cytometry analysis of peripheral blood samples collected from the four experimental groups. Representative gating strategies illustrating the identification of PMN‐MDSCs (CD11b⁺Ly6G⁺Ly6C^−^) and T‐cell subsets (CD3^+^CD4^+^ T cells and CD3^+^CD8^+^ T cells) are shown. Gating strategy for PMN‐MDSCs (CD11b⁺Ly6G⁺Ly6C^−^), CD4^+^ T cells (CD3^+^CD4^+^) and CD8^+^ T cells (CD3^+^CD8^+^). M) Statistical analysis of peripheral blood immune cell frequencies. N–O) Flow cytometry analysis of tumor‐infiltrating PMN‐MDSCs (CD11b⁺Ly6G⁺Ly6C^−^) and T‐cell subsets (CD3^+^CD4^+^ T cells and CD3^+^CD8^+^ T cells) (N). Quantitative analysis is shown (O). P) IHC analysis of CXCR2 and Gr1 expression in subcutaneous tumors derived from the MC38 cell lines Ctrl, sh‐KDM6B, sh‐KDM6B+OE‐SLC10A2, and OE‐SLC10A2. Representative images from each group (n = 5 biological replicates) were shown. The sample size for the (J–O) groups was 5 mice. The data are presented as the means ± SEM and were statistically analyzed by two‐tailed Student's *t* test or one‐way ANOVA. ^*^ *p* < 0.05, ^**^ *p* < 0.01.

We next examined whether SLC10A2 mediates the immunomodulatory effects of KDM6B within the tumor microenvironment. Genetic perturbation experiments in MC38 cells revealed that SLC10A2 plays a central role in regulating immunosuppressive chemokine production. Efficient knockdown of SLC10A2 using two independent shRNAs (sh1 and sh3) (**Figure** **5**A) markedly increased the secretion of CXCL (1, 2, 3, and 5) (Figure 5B), whereas SLC10A2 overexpression significantly suppressed the expression of these chemokines (Figure 5C,D), establishing a clear inverse relationship between SLC10A2 expression and CXCL levels.

Transwell migration assays further demonstrated that SLC10A2 knockdown increased MDSC chemotaxis, whereas SLC10A2 overexpression in KDM6B‐deficient cells (sh‐KDM6B + OE‐SLC10A2) abrogated this effect (Figure 5E,F, Extended Figure 5A). Moreover, the reintroduction of SLC10A2 reversed the upregulation of the expression of MDSC‐associated immunosuppressive markers (S100A8, S100A9, Arg1, TGF‐β, and VEGF‐a) and suppressed AP‐1 activation, as indicated by reduced p‐FOS and p‐JUN levels in KDM6B‐deficient cells (Figure 5G–I). These results suggest that SLC10A2 acts upstream of AP‐1 signaling, functioning as a critical mediator linking KDM6B activity to immune regulation.

To validate these findings in vivo, we used subcutaneous tumor models and assessed the regulatory role of SLC10A2. SLC10A2 restoration (sh‐KDM6B + OE‐SLC10A2) significantly mitigated the accelerated tumor growth driven by KDM6B deficiency (Figure 5J,K). Flow cytometric analysis revealed a substantial reduction in the frequencies of both MDSCs subtypes, especially PMN‐MDSCs, in both peripheral blood and tumor tissues in the SLC10A2 restoration group (sh‐KDM6B + OE‐SLC10A2) compared with those in the sh‐KDM6B group. This reduction was accompanied by a restored proportion of CD4⁺ and CD8⁺ T cells (Figure 5L–O, Extended Figure 5B). Consistent with these findings, immunohistochemical staining confirmed decreased Gr1⁺ and CXCR2⁺ cell infiltration in tumor tissues with SLC10A2 re‐expression (sh‐KDM6B + OE‐SLC10A2) compared with that in the sh‐KDM6B group (Figure 5P). Additionally, we further confirmed that pharmacologic inhibition of KDM6B with GSK‐J4 markedly accelerated tumor growth in wild‐type MC38 models, whereas SLC10A2 overexpression significantly abolished this effect (Figure S7, Supporting Information).

Collectively, these results identify SLC10A2 as a key downstream effector of KDM6B, where its overexpression counteracts the immunosuppressive and protumorigenic consequences of KDM6B loss. These findings underscore the therapeutic relevance of the KDM6B–SLC10A2 axis in maintaining immune equilibrium and restraining colorectal cancer progression.

### 2.6 The KDM6B‐SLC10A2 Axis Governs the CXCL–MDSCs Cascade via ERK/AP‐1 Signaling

**Figure 6:**
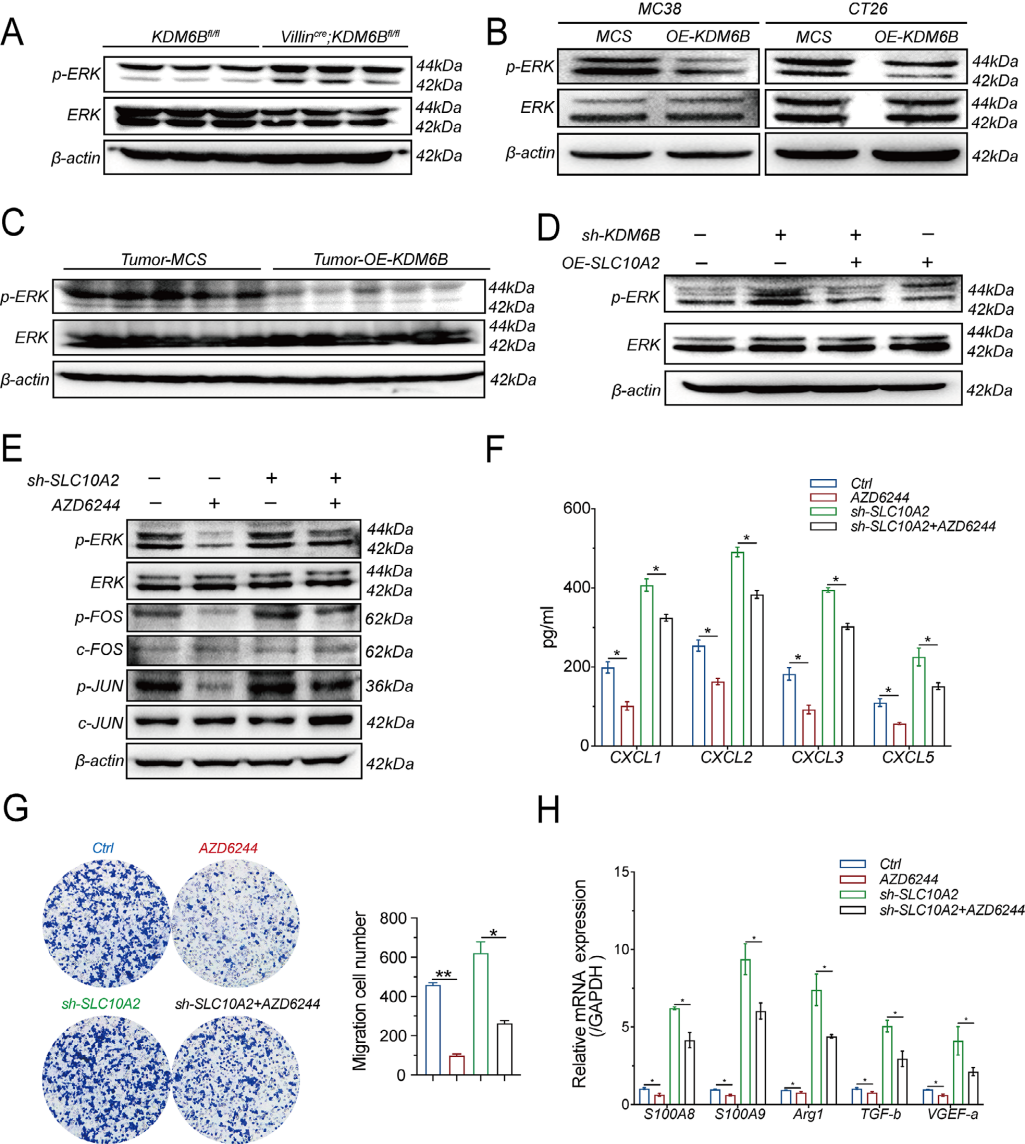
The KDM6B‐SLC10A2 axis governs the CXCL–MDSC cascade via ERK/AP1 signaling. A) Western blot analysis of p‐ERK and ERK expression in colorectal tissues from Villin^Cre^; KDM6B^fl/fl^ mice and KDM6B^fl/fl^ littermate controls after 10 weeks of AOM/DSS treatment. The sample size for each group was n = 3 mice. B) Western blot detection of p‐ERK and ERK in MC38/CT26‐MCS cells and KDM6B‐overexpressing cell lines. β‐actin was used as a loading control. C) Western blot analysis of p‐ERK and ERK in subcutaneous tumors from the MC38‐MCS and MC38‐OE‐KDM6B groups. The sample size for each group was 5 mice. D) Western blot analysis of p‐ERK and ERK in four groups of MC38 cells: Ctrl, sh‐KDM6B, sh‐KDM6B+OE‐SLC10A2, and OE‐SLC10A2. β‐actin was used as a loading control. E) Western blot analysis of p‐ERK, ERK, p‐FOS, c‐FOS, p‐JUN, and c‐JUN in four groups of MC38 cells: Ctrl, AZD6244 (ERK inhibitor), sh‐SLC10A2, and sh‐SLC10A2+AZD6244. β‐actin was used as a loading control. F) ELISA quantification of CXCL (1, 2, 3, and 5) secretion in conditioned media from four groups of MC38 cells: Ctrl, AZD6244, sh‐SLC10A2, and sh‐SLC10A2+AZD6244. G) Transwell migration assay showing MDSC recruitment by conditioned media from four groups of MC38 cells: Ctrl, AZD6244, sh‐SLC10A2, and sh‐SLC10A2+AZD6244. Each group had three biological replicates. H) qPCR analysis of MDSC‐associated immunosuppressive markers (S100A8, S100A9, Arg1, TGF‐β, and VEGF‐a) following stimulation with supernatants from four groups of MC38 cells: Ctrl, AZD6244, sh‐SLC10A2, and sh‐SLC10A2+AZD6244. Data are presented as the means ± SEM, with statistical analyses performed by two‐tailed Student's *t* test or one‐way ANOVA; ^*^ *p* < 0.05, ^**^ *p* < 0.01.

Given that SLC10A2 deficiency accelerates colorectal cancer progression, we next focused on delineating the key signaling nodes downstream of the KDM6B–SLC10A2 axis, with a specific emphasis on the ERK pathway. We performed Western blot analyses in multiple experimental systems to assess ERK signaling modulation. In intestinal epithelium–specific KDM6B knockout mice (Villin^Cre^; KDM6B^fl/fl^), phosphorylated ERK (p‐ERK) levels were markedly increased compared with those in KDM6B^fl/fl^ controls, whereas total ERK levels remained unchanged (**Figure** **6**A). Conversely, KDM6B overexpression in MC38 and CT26 cells resulted in a substantial reduction in p‐ERK levels without affecting total ERK levels (Figure 6B). These findings were recapitulated in subcutaneous tumor models, in which KDM6B overexpression similarly suppressed ERK phosphorylation in vivo (Figure 6C). Collectively, these complementary results establish a robust inverse relationship between KDM6B expression and ERK pathway activation across diverse models.

Rescue experiments further defined SLC10A2 as a critical mediator of this regulatory cascade. In KDM6B‐deficient (sh‐KDM6B) cells, p‐ERK levels were significantly increased, whereas SLC10A2 overexpression (sh‐KDM6B + OE‐SLC10A2) significantly restored ERK phosphorylation to baseline (Figure 6D), indicating that SLC10A2 is both necessary and sufficient to mediate the ERK‐modulatory effects of KDM6B.

To determine whether p‐ERK activity mediates the regulatory effect of the KDM6B/SLC10A2 axis on AP‐1 signaling, we treated SLC10A2‐knockdown MC38 cells with the ERK inhibitor AZD6244. Western blot analysis revealed that SLC10A2 knockdown alone significantly increased the phosphorylation of AP‐1 components (p‐FOS and p‐JUN); notably, this increase was substantially reversed by AZD6244 treatment (Figure 6E). Moreover, this inhibition resulted in consistent phenotypic outcomes across multiple assays: i) a significant reduction in CXCL (1, 2, 3, and 5) secretion (Figure 6F); ii) marked attenuation of MDSC migratory capacity in Transwell assays (Figure 6G); and iii) downregulation of the expression of MDSC‐associated immunosuppressive markers (S100A8, S100A9, Arg1, TGF‐β, and VEGF‐a) at the mRNA level (Figure 6H). These results indicate that ERK activation acts as a downstream mediator of the KDM6B/SLC10A2 axis to activate the AP‐1 pathway.

Together, these findings define a coherent signaling hierarchy in which KDM6B transcriptionally regulates SLC10A2, which in turn modulates ERK/AP‐1 activation to control CXCL chemokine secretion, thereby orchestrating MDSC recruitment and immunosuppressive polarization within the colorectal tumor microenvironment.

### 2.7 Clinical Correlation of the KDM6B‐SLC10A2‐CD33 Axis in Colorectal Cancer Patients

**Figure 7:**
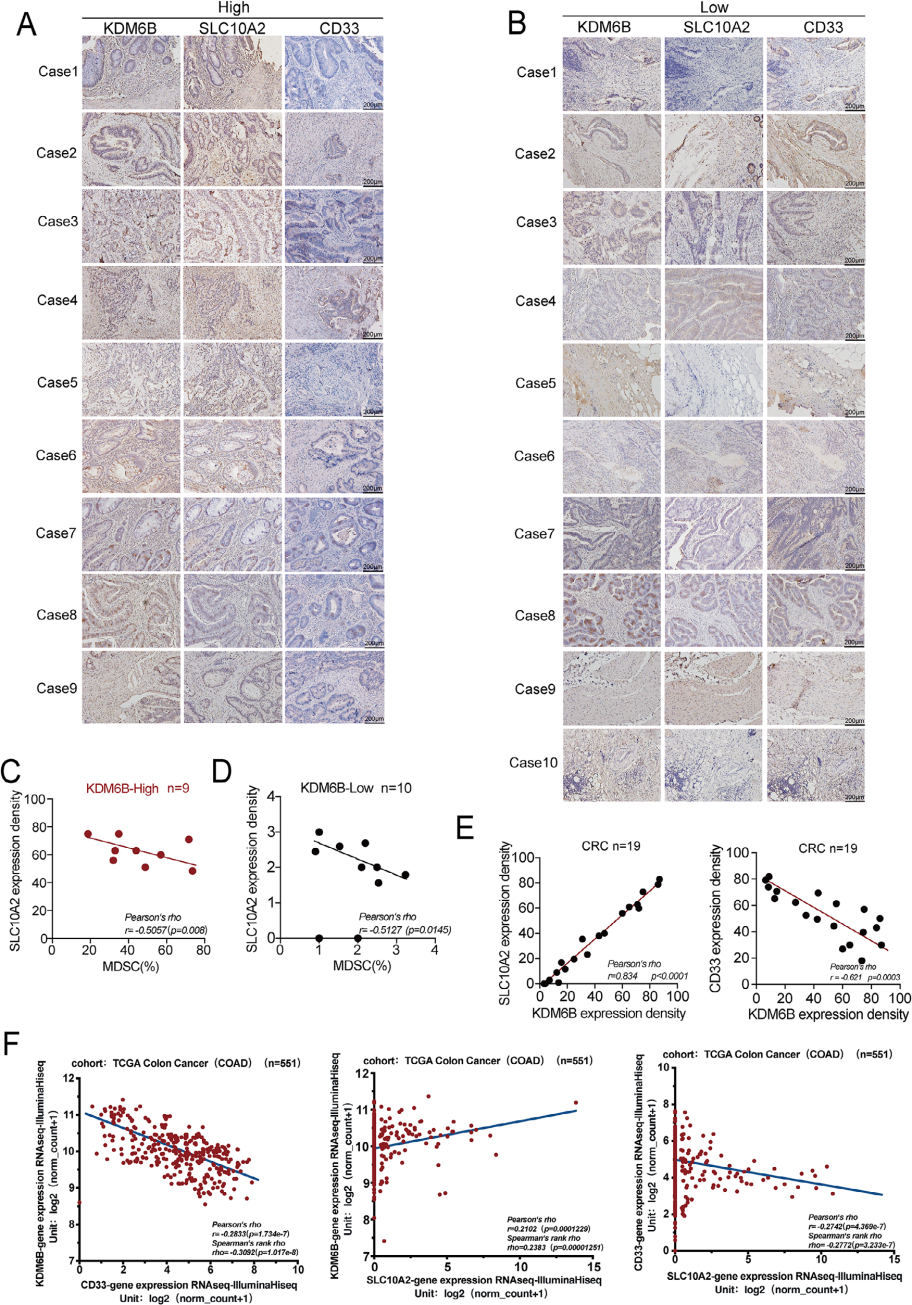
Clinical correlation of the KDM6B‐SLC10A2‐CD33 axis in colorectal cancer patients. A) IHC staining of SLC10A2 and CD33 in tumor tissues from 9 CRC patients with high KDM6B expression (n = 9). B) IHC staining of SLC10A2 and CD33 in tumor tissues from 10 CRC patients with low KDM6B expression (n = 10). C) Scatter plot analysis of SLC10A2 and MDSC (CD33^+^) infiltration levels in KDM6B‐high tumors (n = 9; Pearson's r = −0.5057; p = 0.008). D) Scatter plot analysis of SLC10A2 and MDSC (CD33^+^) infiltration levels in KDM6B‐low tumors (n = 10; Pearson's r = −0.5127; p = 0.0145). E) Correlation analysis of gene expression in 19 CRC samples: Left: SLC10A2 versus KDM6B expression (r = 0.834, *p* < 0.001). Right: CD33 versus KDM6B expression (r = −0.621, p = 0.0003). F) TCGA–COAD cohort analysis (n = 551): Left: KDM6B versus CD33 expression (r = −0.2833, p = 1.734e‐7 (Pearson), p = 1.017e‐8 (Spearman)). Middle: KDM6B versus SLC10A2 expression (r = 0.2102, p = 0.0001229 (Pearson), p = 0.0001229 (Spearman)). Right: CD33 versus SLC10A2 expression (r = −0.2742, p = 4.369e‐7 (Pearson), p = 3.233e‐7 (Spearman)). Group comparisons between KDM6B‐high and KDM6B‐low tumors were analyzed by Pearson's correlation test. Correlation analyses in the human cohort (n = 19) and TCGA cohort (n = 551) were performed using Pearson's correlation test.

To determine the clinical relevance of the KDM6B–SLC10A2 axis, we examined its association with MDSCs infiltration in human colorectal cancer (CRC) specimens. Correlation analyses of KDM6B, SLC10A2, and the MDSC marker CD33 were performed across 19 patient samples, which revealed distinct expression profiles between KDM6B‐high and KDM6B‐low tumors. Immunohistochemical analysis revealed that compared with KDM6B‐low tumors (n = 10), KDM6B‐high tumors (n = 9) exhibited markedly increased SLC10A2 expression and significantly reduced CD33⁺ myeloid cell infiltration (**Figure** **7**A,B). Immunofluorescence costaining for CD11b and CD33 confirmed these findings: KDM6B‐high tumors displayed few CD11b⁺CD33⁺ double‐positive cells, whereas KDM6B‐low tumors exhibited extensive infiltration of such MDSC‐like populations (Extended Figure 7A,B). These combined observations provide strong histological evidence that KDM6B expression inversely correlates with MDSC accumulation in human CRC.

Quantitative correlation analyses further supported these relationships. Within tumor subgroups, there was a significant negative correlation between SLC10A2 expression and CD33 expression in KDM6B‐high samples (Pearson r = −0.5057, p = 0.008), whereas the inverse trend was observed in KDM6B‐low samples (r = −0.5127, p = 0.0145) (Figure 7C,D). Across all 19 cases, KDM6B expression was positively correlated with SLC10A2 expression (r = 0.834; *p* < 0.0001) and negatively correlated with CD33⁺ myeloid infiltration (r = −0.621; p = 0.0003) (Figure 7E). These data align closely with our mechanistic findings in preclinical models, confirming that KDM6B deficiency promotes MDSC recruitment through SLC10A2 downregulation and subsequent increases myeloid cell infiltration in human colorectal cancer tissues.

To evaluate the prognostic implications, we analyzed independent CRC cohorts using multivariable Cox regression models adjusted for clinical covariates. We found that patients with low KDM6B expression had significantly poorer overall survival (log‐rank *p* < 0.0001), and KDM6B remained an independent predictor of adverse outcomes (HR = 10.21, 95% CI 3.19–32.7; *p* < 0.001; C‐index = 0.86) (Extended Figure 7C). Similarly, low SLC10A2 expression correlated with poor survival (log‐rank p = 0.0015) and independently predicted a worse prognosis (HR = 8.62, 95% CI 1.88–39.52; p = 0.006; C‐index = 0.83) (Extended Figure 7D). The other clinical factors followed the expected patterns, with distant metastasis (M1) exerting the strongest adverse effect (Extended Figure 7C,D).

Collectively, these findings establish a clinically relevant link among KDM6B, SLC10A2, and MDSC‐associated CD33 expression, demonstrating that disruption of the KDM6B–SLC10A2 axis increases MDSCs infiltration and contributes to poor prognosis in colorectal cancer patients.

### 2.8 KDM6B Potentiates the Effect of Anti‐PD1 Therapy in Suppressing CRC

**Figure 8:**
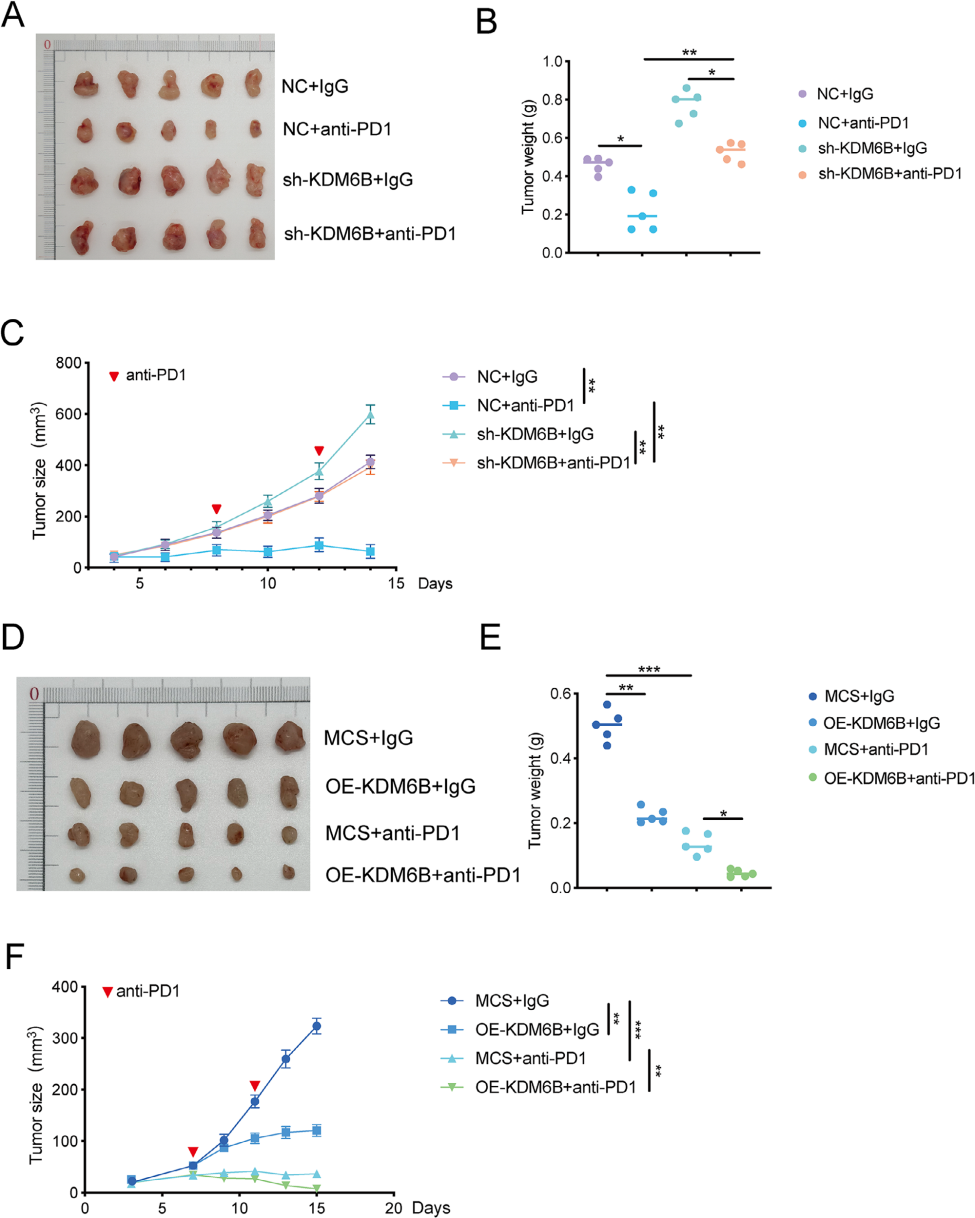
Targeting KDM6B increases the therapeutic efficacy of anti–PD‐1 immunotherapy in MC38 allograft models. A) MC38 cells with KDM6B knockdown (sh‐KDM6B) or negative control (NC) cells were subcutaneously injected into C57BL/6 mice to establish syngeneic transplant tumors, and the mice were subsequently treated with an anti‐PD1 antibody. The experimental groups were as follows: NC+IgG, NC+anti‐PD1, sh‐KDM6B+IgG, and sh‐KDM6B+ anti‐PD1. All the mice (n = 5 per group) were sacrificed on Day 15 post‐injection, and the tumors were harvested for subsequent analysis. B,C) Tumor weight and size of MC38‐NC and MC38‐sh‐KDM6B allografts treated with anti‐PD1 or IgG control. D) MC38 cells overexpressing KDM6B (OE‐KDM6B) or MC38‐MCS control cells were subcutaneously injected into C57BL/6 mice to establish syngeneic transplant tumors, and the mice were then treated with an anti‐PD1 antibody. The experimental groups included MCS+IgG, OE‐KDM6B+IgG, MCS+anti‐PD1, and OE‐KDM6B+anti‐PD1. The mice (n = 5 per group) were sacrificed on Day 20 post‐injection, and the tumors were harvested for subsequent analysis. E,F) Analysis of tumor weight and size from MC38‐MCS and MC38‐OE‐KDM6B allografts treated with anti‐PD1 or IgG control. The data are presented as the means ± SEM. Statistical analyses were performed by two‐tailed Student's *t* test. ^*^ *p* < 0.05, ^**^ *p* < 0.01.

Given our prior findings—both from clinical samples and preclinical models—that low KDM6B expression promotes the recruitment of suppressive MDSCs, thereby fostering an immunosuppressive tumor microenvironment and accelerating CRC progression, we next investigated whether modulating KDM6B expression in CRC could influence the therapeutic efficacy of ICB. To address this, we established subcutaneous allograft mouse models using control (NC) and KDM6B‐knockdown (sh‐KDM6B) MC38 cells and then treated them with an anti‐PD1 antibody. The results showed that compared with the control treatment (NC+ IgG), anti‐PD1 monotherapy effectively reduced tumor volume and weight. Notably, the tumor growth promotion caused by KDM6B knockdown was also reversed by anti–PD1 treatment (**Figure** **8**A–C), indicating that KDM6B deficiency exacerbates immunotherapy resistance.

To further explore whether KDM6B activation could increase anti–PD1 efficacy, we administered an anti–PD1 antibody to control and KDM6B‐overexpressing MC38 subcutaneous allograft models. We found that compared with the control treatment, either KDM6B overexpression alone or anti–PD1 monotherapy significantly reduced tumor volume and weight. Importantly, the combination of KDM6B overexpression and anti–PD1 treatment had the strongest inhibitory effect on MC38 tumor growth, with a 60% reduction in both tumor volume and weight (Figure 8D–F). Collectively, our findings indicate that KDM6B is a potential therapeutic target in colorectal cancer and that its activation not only restores antitumor immunity but also synergizes with anti–PD1 therapy to increase its efficacy, supporting a promising combination strategy.

## 3 Discussion

In this study, we demonstrated that KDM6B depletion significantly increased MDSC accumulation within tumors in multiple animal models, including intestinal epithelium‐specific Villin^Cre^; KDM6B^fl/fl^ mice and syngeneic subcutaneous tumor models. MDSCs represent a heterogeneous population of immune cells that potently suppress antitumor immune responses.^[^ ^16^ ^]^ These cells are abundantly distributed in the peripheral blood and tumor sites of cancer patients, as well as in the spleen and tumor tissues of tumor‐bearing mice.^[^ ^17^ ^]^ Increased MDSC levels are strongly correlated with poor prognosis, disease progression, and decreased response to immunotherapy in patients with breast cancer, colorectal cancer, lung cancer, and hematological malignancies.^[^ ^18^, ^19^, ^20^, ^21^ ^]^ Our findings revealed that KDM6B deficiency in CRC promoted both MDSC accumulation and functional immunosuppression, ultimately leading to reduced T‐cell infiltration and impaired antitumor immunity, thereby underscoring the pivotal role of KDM6B‐induced MDSCs in CRC progression.

To systematically investigate the role of KDM6B in shaping the CRC immune microenvironment, we characterized immune landscapes in both colitis‐associated colon cancer models and subcutaneous tumor models with intestinal epithelium‐specific KDM6B knockout. Our observations revealed that KDM6B deficiency markedly accelerated tumor progression while profoundly remodeling the immune microenvironment. The most striking finding was the dramatic expansion of PMN‐MDSCs across multiple anatomical compartments, including the colonic epithelium, spleen, and bone marrow, in KDM6B‐deficient mice. This expansion coincided with significant reductions in both CD4^+^ and CD8^+^ T‐cell populations, establishing a profoundly immunosuppressive tumor microenvironment. Intriguingly, we detected phenotypic alterations in intestinal macrophages, potentially reflecting MDSC‐mediated polarization effects within the tumor microenvironment.^[^ ^22^ ^]^ Importantly, these findings were validated in clinical datasets, which revealed a significant negative correlation between KDM6B expression and MDSC infiltration in human CRC specimens, along with positive correlations with T‐cell markers. The reciprocal changes in MDSC and T‐cell populations indicate that KDM6B serves as a crucial regulator of the balance between immunosuppressive and immunostimulatory cells in CRC.

On the basis of these observations, we next investigated the molecular mechanisms driving KDM6B‐mediated immune microenvironment remodeling. RNA‐seq analysis of intestinal epithelial cells from KDM6B^fl/fl^; Villin^Cre^ mice revealed significant alterations in inflammatory signaling pathways, with the TNF signaling pathway being the most prominently enriched. Previous studies have demonstrated that the TNF–AP1 signaling axis plays a crucial role in shaping the immune landscape and clinical outcomes in pancreatic cancer.^[^ ^23^ ^]^ Network analysis revealed TNF‐α as the central hub surrounded by key downstream effectors, including the transcription factors Fos and Jun and the chemokines CXCL1, CXCL2, CXCL3, and CXCL5. These findings were validated at both the mRNA and protein levels, demonstrating that KDM6B deficiency activates the TNF–AP1/CXCL axis, with these effects being abolished upon KDM6B overexpression. Given that cytokines play an indispensable role in communication between cancer cells and immune cells,^[^ ^24^ ^]^ the CXCL family of chemotactic factors recruits MDSCs to the tumor microenvironment through binding to the CXCR2 receptor.^[^ ^25^ ^]^ The increased secretion of CXCL chemokines provides a plausible mechanism for the observed MDSC recruitment.

Our subsequent work elucidated the role of the CXCL–CXCR2 axis in MDSC recruitment. Immunohistochemical analysis revealed significantly increased infiltration of CXCR2^+^ and Gr1^+^ cells in KDM6B‐deficient intestinal tissues, an effect that was abolished in subcutaneous tumor models with KDM6B overexpression. Functional migration assays demonstrated that conditioned medium from KDM6B‐deficient MC38 cells increased MDSC recruitment, whereas KDM6B overexpression suppressed this effect. Crucially, CXCR2 blockade markedly reduced KDM6B knockdown‐induced MDSC migration, establishing CXCR2 as the key mediator of the effects of KDM6B on MDSC recruitment. In vivo experiments further supported these findings, showing that KDM6B knockdown promoted tumor growth while increasing PMN‐MDSC proportions and decreasing CD4^+^/CD8^+^ T‐cell infiltration, along with increased CXCR2^+^ and Gr1^+^ cell infiltration in tumor tissues, which were reversed by CXCR2 inhibition. Thus, our study demonstrated that KDM6B drives MDSC accumulation and suppresses T‐cell function in the CRC immune microenvironment, resulting in reduced T‐cell infiltration in KDM6B‐deficient CRC tumors.

We next elucidated the mechanistic link between KDM6B and CXCL secretion. Integrated RNA‐seq and CUT&TAG analyses revealed SLC10A2 as a primary target of KDM6B that promotes CXCL expression in colorectal cancer cells. SLC10A2, an ileal bile acid transporter critical for enterohepatic bile acid‐cholesterol homeostasis,^[^ ^26^ ^]^ is positively correlated with the infiltration of multiple immune cell types (including CD4^+^ and CD8^+^ T cells) and plays important roles in immune microenvironment regulation and clinical prognosis prediction in COAD.^[^ ^27^ ^]^ This transporter activates intracellular signaling pathways, including PKC, PI3K, MAP kinase, and ERK,^[^ ^28^, ^29^ ^]^ with activated ERK propagating signals through the transcription factor AP1.^[^ ^30^ ^]^ Our study demonstrated that KDM6B directly regulates SLC10A2 expression via H3K27me3 demethylation at its promoter. Rescue experiments revealed that SLC10A2 restoration completely reversed KDM6B deficiency‐induced MDSC recruitment and immunosuppression by normalizing ERK/AP1 activation and CXCL secretion, indicating that SLC10A2 is both necessary and sufficient for mediating the immunomodulatory functions of KDM6B. These findings reveal a novel KDM6B–SLC10A2–ERK/AP1–CXCL signaling axis that governs MDSC recruitment in CRC.

Multiple lines of evidence highlight the clinical relevance of our findings. In human CRC specimens, we detected strong positive correlations between KDM6B and SLC10A2 expression, which mirrored our experimental results. Importantly, low expression of either KDM6B or SLC10A2 correlated with increased MDSC infiltration and a poorer patient prognosis, suggesting the potential clinical utility of these genes as prognostic biomarkers. These clinical correlations were further validated in TCGA colon cancer data, which revealed significant negative associations among KDM6B, SLC10A2, and CD33 expression, reinforcing the biological and clinical significance of this regulatory axis. Furthermore, analysis of the TCGA dataset revealed a significant association between KDM6B expression and microsatellite instability (MSI) status. Specifically, KDM6B expression was significantly lower in microsatellite stable (MSS) and MSI‐low (MSI‐L) tumors compared to normal tissues, while its expression was maintained in MSI‐high (MSI‐H) tumors (data not shown). This pattern is particularly relevant as MSI‐H status represents a well‐established biomarker for responsiveness to immune checkpoint blockade. Clinically, ICB benefits only a small subset of CRC patients with high microsatellite instability or mismatch repair deficiency, accounting for ≈5% of metastatic cases. Most patients remain refractory, largely because of an immunosuppressive microenvironment enriched with MDSCs and poor CD8⁺ T‐cell infiltration.^[^ ^31^ ^]^ In this context, our study demonstrated that targeting KDM6B—either via overexpression or in combination with anti‐PD‐1 therapy—markedly suppressed CRC growth in immunocompetent mouse models. Mechanistically, KDM6B targeting reduced CXCL (1, 2, 3, and 5) expression and MDSC infiltration, thereby restoring CD8⁺ T‐cell–mediated antitumor immunity. These findings provides a compelling clinical correlation that further supports KDM6B's role as a modulator of the tumor immune microenvironment, thereby highlighting its promise as a therapeutic target to increase the efficacy of ICI therapy in CRC.

While our study provides substantial new insights into the role of KDM6B in CRC immunobiology, several important questions remain to be answered in future investigations. The precise mechanisms linking SLC10A2 loss to ERK activation require further elucidation, as does the potential crosstalk between KDM6B and other epigenetic regulators in shaping the tumor immune microenvironment. Additionally, developing more sophisticated genetic models, such as intestine‐specific KDM6B/SLC10A2 dual knockout mice, will be crucial for further in vivo validation of these findings and exploration of potential therapeutic interventions.

**Figure 9:**
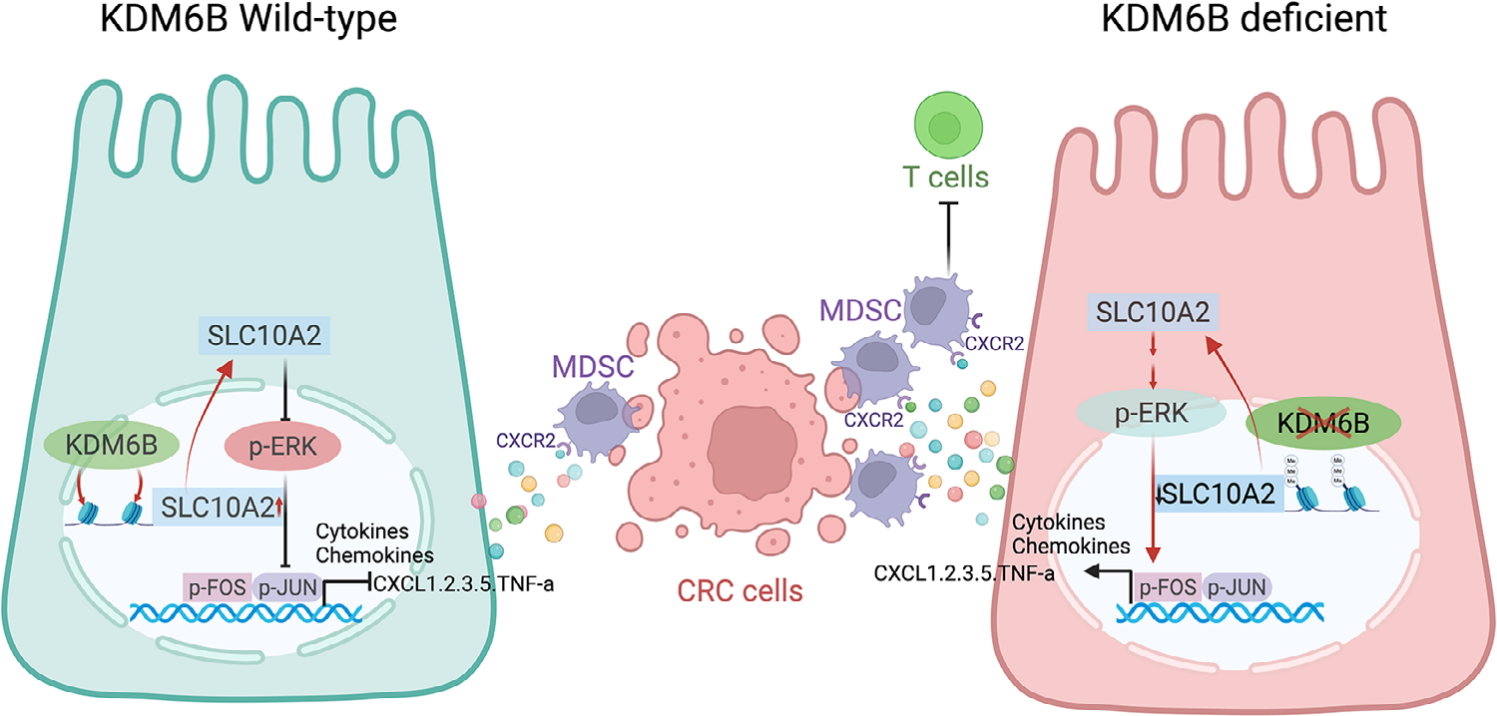
Schematic of the mechanism of KDM6B‐driven immunosuppression in colorectal cancer via the SLC10A2/CXCL axis. (Created with bioRender.com, with permission.). In wild‐type intestinal epithelial cells, KDM6B demethylates SLC10A2 to promote its expression, after which SLC10A2 suppresses ERK phosphorylation, inhibiting AP‐1 (Fos/Jun) transcription and chemokine production. In KDM6B‐deficient intestinal epithelial cells, SLC10A2 methylation increases, and its expression decreases. Phosphorylated ERK promotes transcription factor AP‐1 (Fos/Jun) activation and chemokine production, leading to increased MDSCs recruitment, increased T‐cell suppression, and accelerated colorectal cancer progression.

In conclusion, our study elucidates how KDM6B deficiency promotes an immunosuppressive tumor microenvironment in CRC through a novel KDM6B–SLC10A2–ERK/AP‐1–CXCL signaling axis (**Figure** **9**). These findings not only advance our fundamental understanding of CRC immunobiology but also identify new therapeutic opportunities for targeting MDSC‐mediated immune evasion in CRC. The comprehensive nature of our experimental approach, spanning molecular mechanisms to clinical correlations, provides a robust foundation for future translational studies aimed at improving outcomes for CRC patients.

## 4 Experimental Section

### Cell Lines and Cell Culture

The murine CRC cell lines CT26 (RRID: CVCL_7254) and MC38 (Ubigene Cat# YC‐A002, R RID: CVCL_B288) were purchased from the American Type Culture Collection (Manassas, VA). All cells were cultured in Dulbecco's modified Eagle's medium supplemented with 10% fetal bovine serum (Thermo Scientific, Waltham, MA) and maintained at 37 °C in a humidified incubator with 5% CO_2_.

### Cell Line Authentication and Mycoplasma Testing

Cell lines were authenticated using short tandem repeat (STR) profiling (performed by Genetic Testing Biotechnology Corporation (Suzhou, China) upon receipt and every 6 months during the study to ensure genetic stability and the absence of cross‐contamination. All the cell lines were routinely tested for mycoplasma contamination every month using the MycoAlert Mycoplasma Detection Kit (Lonza, Basel, Switzerland; catalog no. LT07‐118). The cells were confirmed to be mycoplasma free throughout the study. The results were documented in laboratory records.

### Vector Construction and Establishment of Stable Cell Lines

The DNA sequence encoding mouse KDM6B was amplified from the pCMV‐HA‐KDM6B plasmid (Addgene, Cambridge, MA) and cloned and inserted into the pLV‐H1‐EF1α‐puro vector (Biosettia, San Diego, CA); the coding sequence of mouse SLC10A2 was similarly amplified and cloned, and inserted into the pCDH‐CMV‐MCS‐EF1‐Puro vector (Biosettia). For gene silencing, short hairpin RNA (shRNA) sequences targeting KDM6B were cloned and inserted into the pLV‐EF1α‐MCS‐IRES‐Bsd vector (Biosettia), and shRNAs targeting SLC10A2 were cloned and inserted into the pLKO.1‐MCS‐puro vector (Biosettia, San Diego, CA). Lentiviruses carrying the overexpression constructs, gene‐silencing vectors, or empty vectors were produced according to the manufacturer's protocol. To establish stable recombinant cell lines, cells were transduced with lentivirus‐containing medium supplemented with 8 µg mL^−1^ polybrene for 48 h, followed by selection with either 10 µg mL^−1^ blasticidin or 1 µg mL^−1^ puromycin for one week.

### Tumor Models

KDM6B‐knockout or control MC38 cells were subcutaneously injected into the dorsal region of immunocompetent male C57BL/6 mice aged 4–6 weeks. The CXCR2 antibody SB‐225002 (MCE; Cat# HY‐16711) or phosphate‐buffered saline was administered via intraperitoneal injection at a dose of 2 mg kg^−1^ daily. The tumor size was measured every other day.

The tumorigenesis procedure was performed according to a previously reported method.^[^ ^15^ ^]^ Briefly, 6‐week‐old mice (15 mice per group) were intraperitoneally injected with AOM dissolved in 0.9% NaCl at a dose of 12.5 mg kg^−1^ body weight and then challenged with three cycles of 1.5% (w/v) DSS in the drinking water for 7 days. The first cycle began immediately after AOM injection, followed by a recovery period with normal drinking water for 2 weeks before the second cycle was initiated. Two weeks after the final DSS treatment, the mice were sacrificed. All of the colon tumor analyses were conducted under a stereomicroscope.

### Ethical Approval for Animal Experiments

All animal experiments were conducted in accordance with the National Institutes of Health (NIH) Guide for the Care and Use of Laboratory Animals and were approved by the Animal Ethics Committee of NanKai Hospital (GENINK‐20240052). All efforts were made to minimize animal suffering and reduce the number of animals used. Housing conditions (temperature, humidity, and light/dark cycles) and euthanasia methods (euthanasia was completed by cervical dislocation after induction of deep anesthesia with isoflurane) were strictly followed as per IACUC guidelines.

### Isolation of Single Cells From Mouse Blood, Intestinal Tissue, Immune Organs, and Tumors

Single‐cell suspensions were prepared from multiple tissue sources using standardized protocols. Peripheral blood samples were collected via the retro‐orbital venous plexus into EDTA‐coated tubes (BD Biosciences). Splenocytes were obtained by mechanical dissociation of the spleens on ice followed by filtration through 70‐µm cell strainers (Corning). Bone marrow cells were harvested by flushing femurs and tibiae with cold PBS using a 25‐gauge needle. For tumor and intestinal tissues, specimens were minced into <1 mm^3^ fragments and digested for 30 min at 37 °C with an enzymatic cocktail containing collagenase IV, hyaluronidase, and DNase I (0.05 mg mL^−1^ each; Sigma‒Aldrich). Following digestion, the cell suspensions were filtered through 70‐µm strainers and treated with ACK lysis buffer (Solarbio, Cat# R1010) for erythrocyte removal. Tumor‐infiltrating myeloid cells were further enriched by Ficoll‒Paque PLUS (GE Healthcare) density gradient centrifugation. For flow cytometry analysis, cells were washed twice with cold PBS containing 2% FBS and stained with fluorochrome‐conjugated antibodies for 30 min at 4 °C, maintaining >90% viability as determined by Trypan blue exclusion throughout all procedures.

### Flow Cytometry and Gating Strategy

Single‐cell suspensions from the indicated tissues were prepared as described above. Single cells were resuspended in PBS containing 1% BSA and stained with antibodies for 30 min at room temperature in the dark. The cells were then analyzed using an EXFLOW flow cytometer (Dakewe, Beijing, China), and the data were analyzed using FlowJo software (Tree Star, Inc., Ashland, OR). Antibodies against the following proteins were used: CD45 (E‐AB‐F1136E; Elabscience Biotechnology, Wuhan, China), CD11b (E‐AB‐F1081F; Elabscience), Ly6G (E‐AB‐F1108D; Elabscience), Ly6C (E‐AB‐F1121H; Elabscience), CD3 (E‐AB‐F1013C; Elabscience), CD4 (E‐AB‐F1097F; Elabscience), CD8 (E‐AB‐F1104D; Elabscience), F4/80 (E‐AB‐F0995H; Elabscience), CD86 (E‐AB‐F0994D; Elabscience), and CD206 (E‐AB‐F1135C; Elabscience). Single‐cell populations were first screened by forward scatter (FSC) and side scatter (SSC), followed by identification of myeloid cells on the basis of CD11b^+^ labeling, and then, PMN‐type MDSCs were identified by Ly6G^+^Ly6C^−^ labeling, while M‐type MDSCs were identified by Ly6G^−^Ly6C^+^ labeling. CD4^+^ T cells were identified by CD3^+^CD4^+^ labeling, while CD8^+^ T cells were identified by CD3^+^CD8^+^ labeling; M1‐type macrophages were identified by F4/80^+^CD86^+^ labeling, whereas F4/80^+^CD206^+^ labeling identified M2‐type macrophages. For analysis of T cells and MDSCs, cells were first gated by FSC/SSC to exclude debris and doublets, followed by live/dead discrimination using viability dyes. Isotype controls were included to determine background fluorescence. Sequential gating was applied to identify specific cell populations. The detailed gating procedures are provided in Figure S1A,B (Supporting Information).

### CUT&TAG Sequencing

CUT&TAG assays were performed using the Hyperactive In‐Situ ChIP Library Prep Kit (Illumina) to map H3K27me3 modifications throughout the genome. Briefly, cells were bound to concanavalin A‐coated magnetic beads and permeabilized with digitonin to enable antibody access. Sequential incubations were carried out with 1) an anti‐H3K27me3 primary antibody (ab6002; Abcam), 2) a species‐matched secondary antibody, and 3) a hyperactive pA‐Tn5 transposase. The pA‐Tn5 complex simultaneously cleaves DNA at protein binding sites and ligates Illumina adapters (P5/P7) in situ. After PCR amplification with adapter‐specific primers, library quality was verified using an Agilent 2100 Bioanalyzer before 150 bp paired‐end sequencing on the Illumina NovaSeq 6000 platform.

### RNA Sequencing Library Construction and Data Processing

Total RNA was isolated using TRIzol reagent (Thermo Fisher Scientific, 15596018), and its integrity was verified by agarose gel electrophoresis. Sequencing libraries were constructed as follows: First‐strand cDNA was synthesized from qualified RNA samples using random hexamer primers and M‐MuLV Reverse Transcriptase. Second‐strand cDNA synthesis was subsequently performed using DNA polymerase I and RNase H. The resulting cDNA fragments were end‐repaired, A‐tailed, and ligated with Illumina RNA Index Adapters. After PCR amplification, the final libraries were quantified using a Qubit 2.0 fluorometer (Life Technologies), and their size distribution was assessed on an Agilent 2100 Bioanalyzer. Paired‐end sequencing (PE150) was conducted on an Illumina NovaSeq 6000 platform by Novogene Bioinformatics Technology Co., Ltd. (Beijing, China), which yielded ≈12 to 15 million raw reads per sample. For data processing, the clean reads were aligned to the reference genome using HISAT2 (version 2.2.1). Transcript assembly and abundance estimation were performed with StringTie, and differential expression analysis was carried out using both EdgeR (version 4.6.2) and DESeq2 (version 1.48.1) software. Functional enrichment analysis of Gene Ontology (GO) terms and Kyoto Encyclopedia of Genes and Genomes (KEGG) pathways was conducted using the ClusterProfiler R package (version 4.2.1). Gene set enrichment analysis (GSEA) was performed using GSEA software (version 4.3.X).

### Chromatin Immunoprecipitation (ChIP) Assay

Chromatin immunoprecipitation (ChIP) assays were performed using a ChIP Assay Kit (Beyotime, Cat# P2078, China) following the manufacturer's protocol. DNA‒protein complexes were crosslinked and extracted from MC38 cells, followed by chromatin fragmentation and purification using a DNA Cleanup Kit (Beyotime, Cat# D0033, China). The samples were then incubated overnight at 4 °C with rotation with either an anti‐H3K27me3 antibody (ab6002; Abcam) or normal rabbit IgG as a negative control, followed by capture with Protein A/G agarose beads and extensive washing. The enriched DNA was then analyzed by PCR amplification using two primer sets on the SLC10A2 promoter sequence: Primer 1, forward: 5′‐TGTGAGGGGCATGATTCCAA‐3′, reverse: 5′CATGGGATGCAACGTGGAAA‐3′; Primer 2, forward: 5′‐ACTGACAGAGGAAGCCAACA‐3′, reverse: 5′‐GTGGTCCTAAGTACGGTGCT‐3′.

### Real‐Time Quantitative PCR

Total RNA was extracted from peritoneal metastatic tumor tissue and cells using TRIzol (Invitrogen) reagent following the specified protocol. Subsequently, 2 µg of RNA was reverse transcribed into cDNA with a HifairIII 1st Strand cDNA synthesis kit (Yeasen Biotech) following the manufacturer's instructions. The qPCR procedure used the HieffqPCR SYBR Green Master Mix Reagent (Yeasen Biotech) and was conducted under manufacturer‐specified conditions. SYBR Green‐based qPCR was used to amplify primers for various markers. The mRNA levels were normalized to those of GAPDH (ΔCt = Ct gene of interest − Ct GAPDH) and are presented as relative mRNA expression (ΔΔCt = 2− (ΔCt sample − ΔCt control)) or as fold change values.

### Western Blotting Analysis

Colonic tissues, tumor tissues, and cells were homogenized in RIPA buffer (Yeasen Biotech, Shanghai, China) supplemented with a protease and phosphatase inhibitor cocktail (Yeasen Biotech, Shanghai, China). Protein concentrations were determined using the Pierce BCA Protein Assay Kit (Thermo Fisher Scientific, USA). Approximately 30 µg of protein was resolved by SDS‒PAGE and electrotransferred onto polyvinylidene fluoride (PVDF) membranes (Bio‐Rad, Shanghai, China). The membranes were blocked with 5% nonfat milk for 1 h at room temperature, followed by overnight incubation with primary antibodies at 4 °C. Horseradish peroxidase (HRP)‐conjugated secondary antibodies (ZSGB‐BIO, Beijing, China) were subsequently applied, and specific protein bands were visualized using an enhanced chemiluminescence (ECL) Western blotting detection system (Millipore, USA). The primary antibodies used were as follows: antibodies against KDM6B (#3457), ERK (#4695), p‐ERK (#9102), c‐JUN (#9165), and p‐JUN (#3270) were obtained from Cell Signaling Technology (Danvers, MA, USA), and antibodies against β‐actin (AC026), SLC10A2 (#25245‐1‐AP), c‐FOS (#64 693), p‐FOS (#66590‐1‐Ig), TNF‐a (#17590‐1‐AP), and H3k27me3 (#91 167) were obtained from Proteintech in Wuhan, China.

### MDSCs Isolation and Migration Assay

Eight‐week‐old C57BL/6 mice were euthanized by cervical dislocation, and bone marrow cells were flushed from the femurs and tibiae using sterile syringes. The isolated bone marrow cells were cultured in Dulbecco's modified Eagle's medium supplemented with 10% fetal bovine serum (Gibco, USA). For MDSCs differentiation, the cell suspension was adjusted to a concentration of 3 × 10^5^ cells mL^−1^ in RPMI medium containing GM‐CSF (10 ng mL^−1^) and IL‐6 (10 ng mL^−1^), after which the cells were seeded into 24‐well flat‐bottom plates and cultured for 4 days in a CO_2_ incubator (37 °C, 5% CO_2_). For migration assays, MDSCs (1 × 10^5^ cells/well) were plated in the upper chamber of a Transwell system (8 µm pore size) with 100 µL of RPMI medium, while 600 µL of serum‐free conditioned medium collected from either CT26 or MC38 cells was added to the lower chamber. After 4 h of incubation, the number of migrated cells in the lower chamber was quantified.

### Enzyme‐Linked Immunosorbent Assay (ELISA)

The concentrations of inflammatory cytokines (CXCL1, CXCL2, CXCL5, and TNF‐α) in cell culture supernatants and mouse serum were quantified using commercial ELISA kits (Tong Wei, Shanghai, China) according to the manufacturer's protocols.

### Immunohistochemical (IHC) Staining

Formalin‐fixed, paraffin‐embedded tissue sections were deparaffinized using xylene and a graded ethanol series, followed by antigen retrieval in citrate buffer (pH 6.0; Solarbio Science & Technology, Beijing, China). The sections were then treated with 3% hydrogen peroxide to quench endogenous peroxidase activity and blocked with 5% goat serum. Primary antibody incubation was performed overnight at 4 °C, followed by sequential incubations with biotinylated secondary antibody and streptavidin‐peroxidase complex. Immunocomplexes were visualized using 3,3′‐diaminobenzidine (DAB) as the chromogenic substrate, with hematoxylin counterstaining for nuclear visualization. Images were acquired using an inverted microscope (Olympus Co., Tokyo, Japan). Primary antibodies against the following proteins were used: KDM6B (ab38113; Abcam), CD33 (17425‐1‐AP; Proteintech), SLC10A2 (25245‐1‐AP; Proteintech), CXCR2 (19538‐1‐AP; Proteintech), Gr1 (cat. no.: CL488‐65140), and Ki67 (ab15580; Abcam).

### Immunofluorescence Staining

The cells were fixed with methanol at 4 °C for 15 min, followed by blocking with 1% BSA for 1 h at room temperature. The cells were then incubated with primary antibodies overnight at 4 °C and subsequently with secondary antibodies for 1 h at room temperature. Nuclear DNA was counterstained with 4′,6‐diamidino‐2‐phenylindole (DAPI). Fluorescence images were acquired using a Leica fluorescence microscope (Leica Microsystems, Germany).

### Human CRC Specimens

Colorectal cancer (CRC) tissue specimens were obtained from Nankai Hospital with informed consent from all patients and the Clinical Trial Ethics Committee of Tianjin Nankai Hospital (approval no.: NKYY_YX_IRB_2018_039_01, valid from January 2019 to January 2024). All the tissues were immediately fixed with 4% formalin. This study complied with ethical guidelines, and all the data were anonymized for analysis. Immunohistochemical staining was performed on paraffin sections using antibodies against KDM6B (ab38113; Abcam), CD33 (17425‐1‐AP; Proteintech), and SLC10A2 (25245‐1‐AP; Proteintech). The KDM6B expression score (positive cell percentage × staining intensity) was evaluated by two independent pathologists following standard protocols. All procedures complied with ethical requirements.

### Statistical Analysis

All the statistical analyses were performed using GraphPad Prism software (version 9.0). Data preprocessing: Prior to analysis, the normality of all the data distributions was assessed using the Shapiro‒Wilk test, and the homogeneity of variances was confirmed using Levene's test. Data presentation: Continuous data are presented as the mean ± standard error of the mean (SEM). Sample size: The sample size (n) for each experiment, representing independent biological replicates, is provided in the corresponding figure legend. Statistical methods: Based on the pre‐analysis results, normally distributed data were analyzed using parametric tests. Two‐group comparisons were performed using an independent Student's t‐test after assessing variance homogeneity with Levene's test; when variances were unequal, a corrected t′‐test was applied. Comparisons among three or more groups were conducted using one‐way or two‐way analysis of variance (ANOVA), as appropriate for the experimental design. For longitudinal measurements of mouse body weight, two‐way ANOVA with Tukey's post hoc test was used to identify specific differences over time and between groups. For data that did not meet the assumptions of normality or homoscedasticity, the nonparametric Mann‒Whitney U test (for two groups) was used. The threshold for statistical significance was set at a two‐sided alpha level of 0.05 for all tests. p values are shown as ^*^ *p* < 0.05, ^**^p <0.01, and ^***^ *p* < 0.001.

### Ethics Approval

Animal experiments were conducted under the guidance of the Animal Care and Use Committee of Tianjin Nankai Hospital (GENINK‐20240052). CRC tissue specimens were obtained from Nankai Hospital with informed consent from all patients and the Clinical Trial Ethics Committee of Tianjin Nankai Hospital (Approval No: NKYY_YX_IRB_2018_039_01).

## Conflict of Interest

The authors declare no conflict of interest.

## Author Contributions

Z.H. and J.X. contributed equally to this work. Z.H. performed to the methodology, investigation, formal analysis, and writing of the original draft. J.X. performed to the validation, formal analysis, funding acquisition, and writing of the original draft. B.L. performed to the methodology and visualization. X.J. performed to the methodology, formal analysis, and visualization. Y.H. performed to the methodology and visualization. H.Y., Q.G., and R.G. performed to the methodology. A.Z., D.Z., and S.Y. acquired funding. D.L., X.W., and T.L. supervised the project. X.W. provided resources and supervision. X.Y. conceptualized, supervised, and reviewed and edited the manuscript, and acquired funding. Q.Z. conceived the project, administered the project, wrote–reviewed and edited the manuscript, and acquired funding.

## Supporting information



Supporting Information

## Data Availability

The data that support the findings of this study are available from the corresponding author upon reasonable request.
