# Supplementary material for: The KDM6B/SLC10A2 Axis Suppresses MDSCs Recruitment via ERK/AP‐1 Signaling in Colorectal Cancer
Source: Adv Sci (Weinh). 2025 Dec 12;13(9):e14086. doi: 10.1002/advs.202514086 (PMC12903986; doi:10.1002/advs.202514086)
Supplement: Supplementary file 1 — Supporting Information [file ADVS-13-e14086-s001.docx]

**Extended Figures**


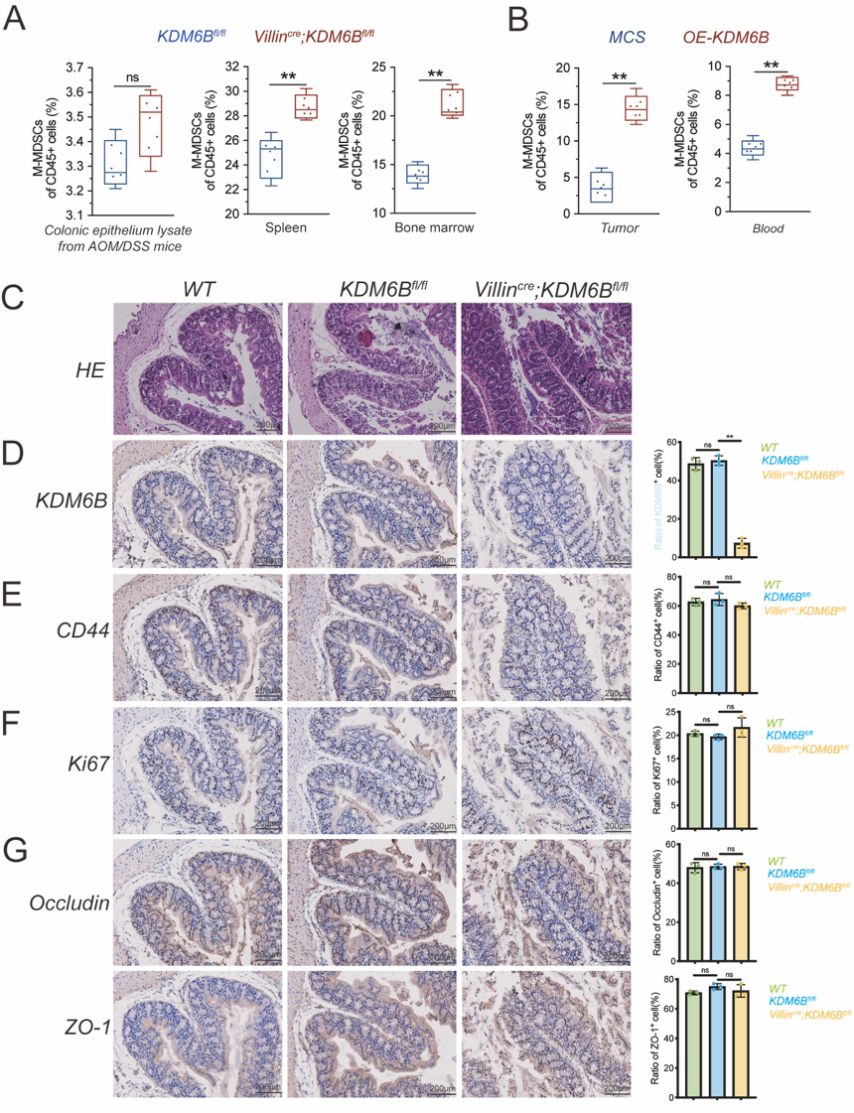


**Extended Figure 1. Association of epithelial KDM6B with intestinal homeostasis and immune infiltration**

(A) In the AOM/DSS model of colitis‑associated tumorigenesis, flow cytometry quantifies M‑MDSCs (CD11b^+^Ly6C^+^Ly6G^−^) in spleen and bone marrow from KDM6B^fl/fl^ and Villin^Cre^; KDM6B^fl/fl^ mice; epithelial lysates show the proportion of M‑MDSCs among CD45^+^ cells. Statistics: unpaired two‑sided t test; n=5 per group (spleen, bone marrow, and epithelial lysates).

(B) In tumor‑bearing mice, proportions of M‑MDSCs in tumors and peripheral blood comparing control (MCS) and epithelial KDM6B overexpression (OE‑KDM6B). Statistics: unpaired two‑sided t test; n=5 per group (tumor and blood).

(C–G) Representative histology and IHC on paraffin sections: H&E (C), KDM6B (D), CD44 (E), Ki67 (F), and tight junction proteins Occludin and ZO‑1 (G). Quantifications at right. n=3 mice per group; ≥3 non‑overlapping fields per mouse. Statistics: one‑way ANOVA with Tukey’s multiple comparisons. Scale bars as indicated. ns, not significant; * P<0.05; **P<0.01.


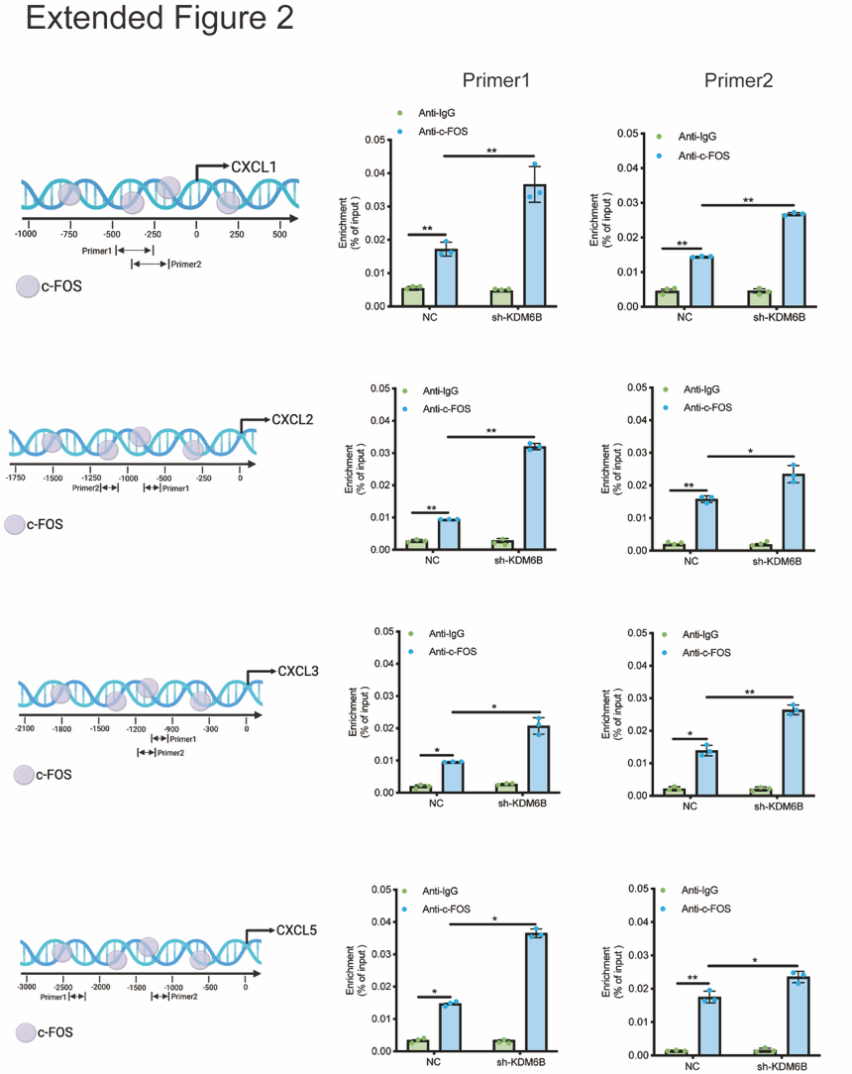


**Extended Figure 2. KDM6B loss increases c‑FOS occupancy at chemokine promoters**

ChIP‑qPCR schematics and enrichment at two primer regions (Primer1/Primer2) within CXCL1, CXCL2, CXCL3, and CXCL5 promoters. IgG serves as control; NC (control cells) versus KDM6B‑silenced cells (sh‑KDM6B). Enrichment is presented as % input. Statistics: two‑way ANOVA (factors: treatment [NC vs sh‑KDM6B] and antibody [IgG vs anti‑c‑FOS]) followed by Tukey’s correction; n=3 independent biological replicates. ns, not significant; * P<0.05; ** P<0.01.


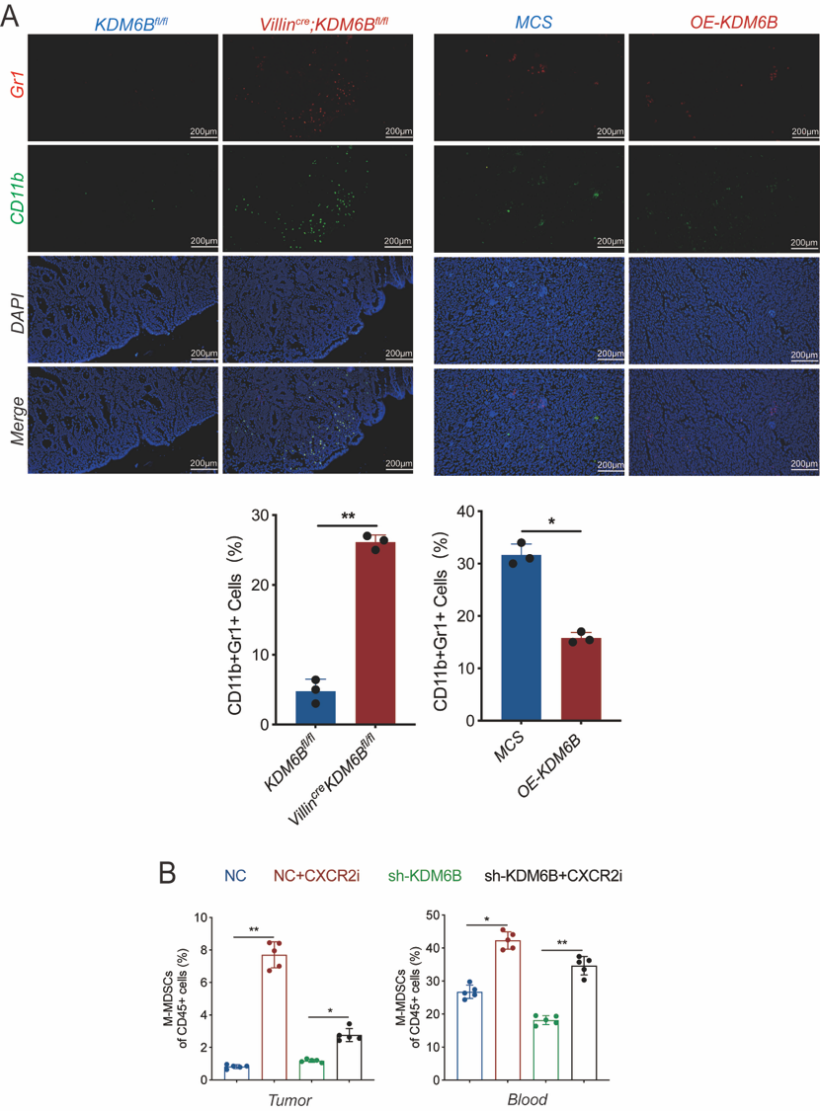


**Extended Figure 3. KDM6B modulates intratumoral MDSCs recruitment**

(A) Dual IF on tumor cryosections for CD11b and Gr1 (DAPI counterstain) comparing KDM6B^fl/fl^ versus Villin^Cre^; KDM6B^fl/fl^, and MCS versus OE‑KDM6B tumors. Quantification of CD11b^+^Gr1^+^ cells is averaged from ≥3 fields per mouse; n=3 per group. Statistics: unpaired two‑sided t test. Scale bars as indicated. ns, not significant; * P<0.05; ** P<0.01.

(B) M‑MDSCs proportions in tumors and blood across four conditions: NC, NC plus CXCR2 inhibitor (CXCR2i), sh‑KDM6B, and sh‑KDM6B plus CXCR2i. Statistics: one‑way ANOVA with Tukey’s post hoc test; n=5 per group.


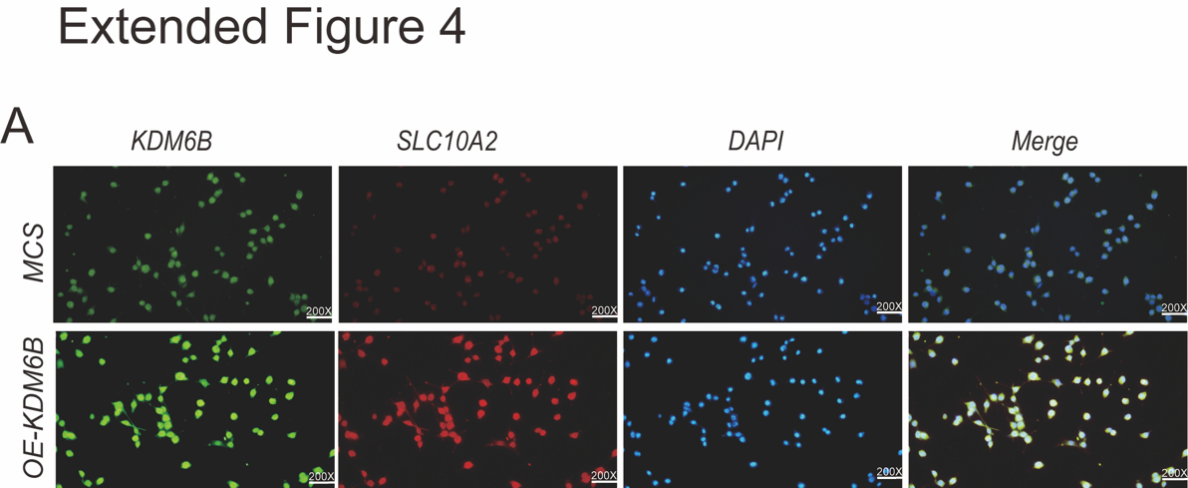


**Extended Figure 4. KDM6B upregulation coincides with increased SLC10A2**

(A) IF co‑localization in MC38 cells showing enhanced SLC10A2 signal upon KDM6B overexpression (DAPI counterstain). Representative of three independent experiments. Scale bars as indicated.


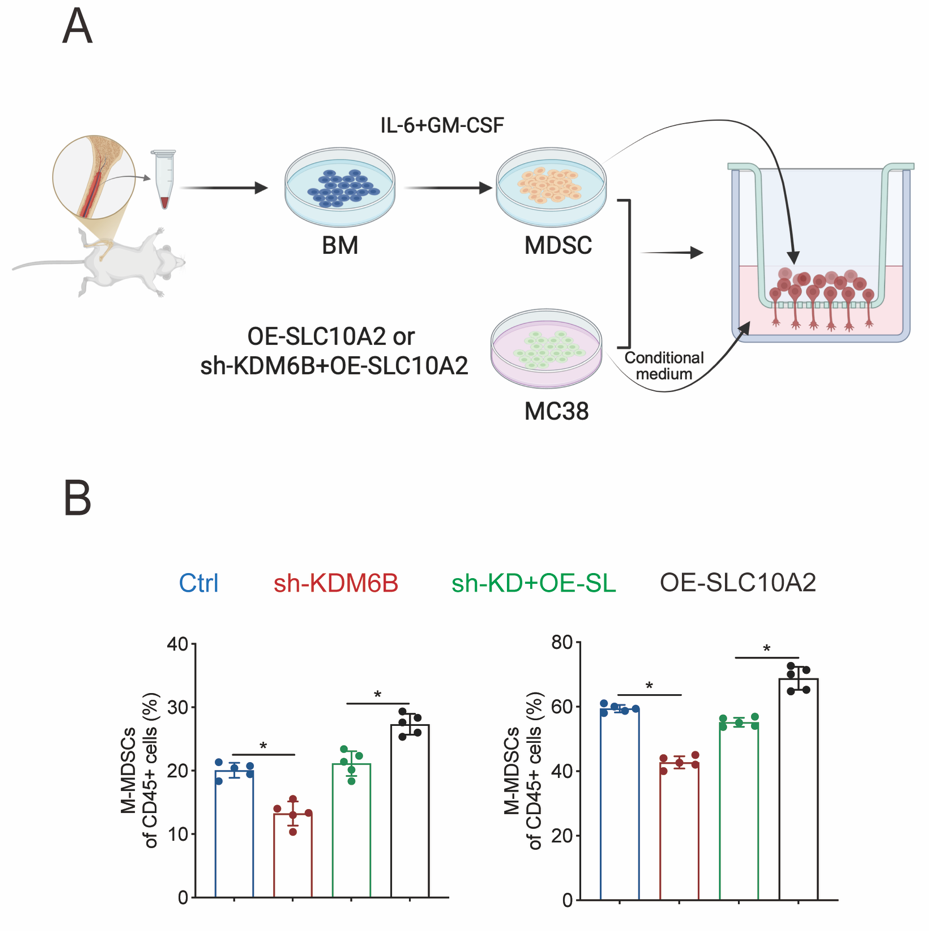


**Extended Figure 5. SLC10A2 acts KDM6B‑dependent generation of M‑MDSCs**

(A) Experimental workflow: mouse bone marrow cells were treated with GM-CSF (20 ng/mL) and IL-6 (20 ng/mL) for 4 days to induce into MDSCs; conditioned media collected from MC38 cells (Ctrl, sh‑KDM6B, sh‑KDM6B +OE‑SLC10A2, or OE‑SLC10A2.) were applied to the induction system. Schematic for illustration only; not analyzed statistically.

(B) M‑MDSCs proportions in tumors and blood across four conditions: Four groups: Ctrl, sh‑KDM6B, sh‑KDM6B plus OE‑SLC10A2 (sh‑KD+OE‑SL), and OE‑SLC10A2. Two independent experimental panels are shown (left, right). Statistics: one‑way ANOVA with Tukey’s multiple comparisons; n=5 biological replicates per group. Quantification shown as mean ± SEM. Significance symbols: * P<0.05.


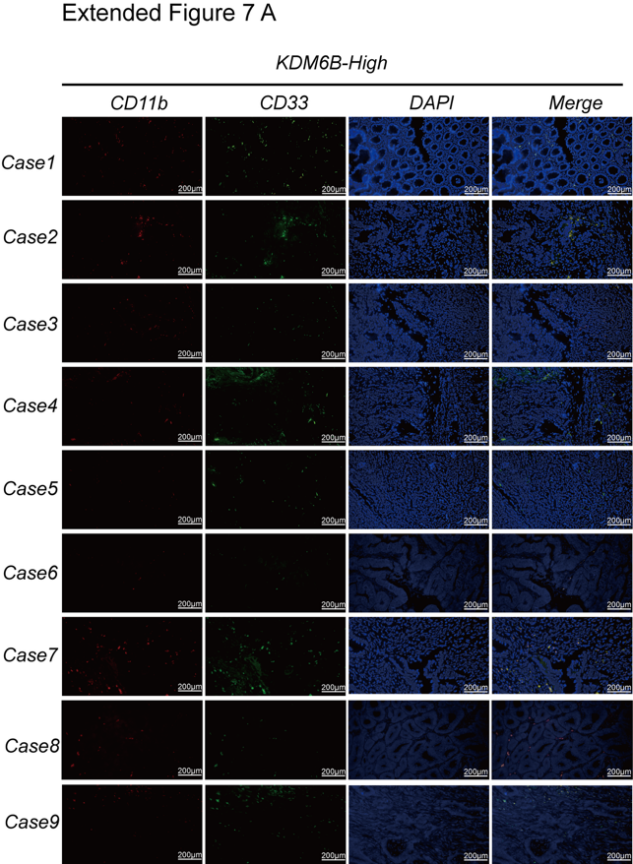

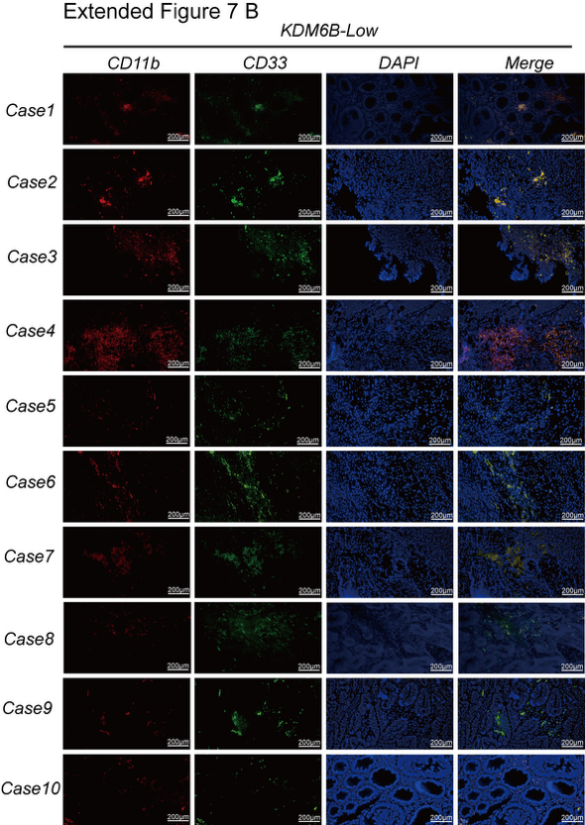


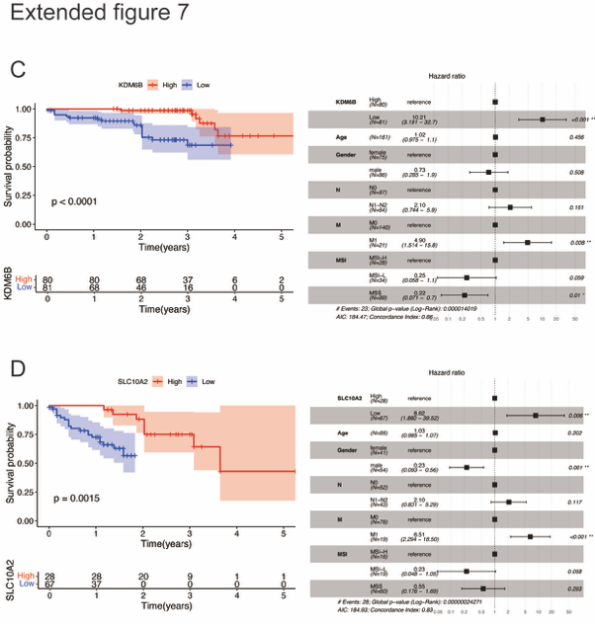


**Extended Figure 7. The correlation between the expression of KDM6B/SLC10A2 and the infiltration of MDSCs as well as prognosis in human colorectal cancer**

(A–B) Multiplex IF on human CRC tissue sections stratified by KDM6B expression: KDM6B‑High (A) and KDM6B‑Low (B); representative fields at 200× for CD11b and CD33 with DAPI. At least three fields per case; cases: KDM6B‑High n=9, KDM6B‑Low n=10. Images are representative; quantitative data are provided in the main or extended datasets.

(C) Kaplan–Meier overall survival (OS) by KDM6B High/Low groups with multivariable Cox proportional hazards models (covariates: age, gender, N, M, MSI). Statistics: Log‑rank test for KM curves (log-rank p < 0.0001); Cox models report HR = 10.21, 95% CI 3.19–32.7, p < 0.001. Sample sizes were n=161.

(D) Kaplan–Meier overall survival (OS) by SLC10A2 High/Low groups with multivariable Cox proportional hazards models (covariates: age, gender, N, M, MSI). Statistics: Log‑rank test for KM curves (log-rank p= 0.0015); Cox models report HR =8.62, 95% CI 1.88–39.52, p = 0.006; C-index = 0.83. Sample sizes were n=95.

**Supplemental Figures and Figure legends**


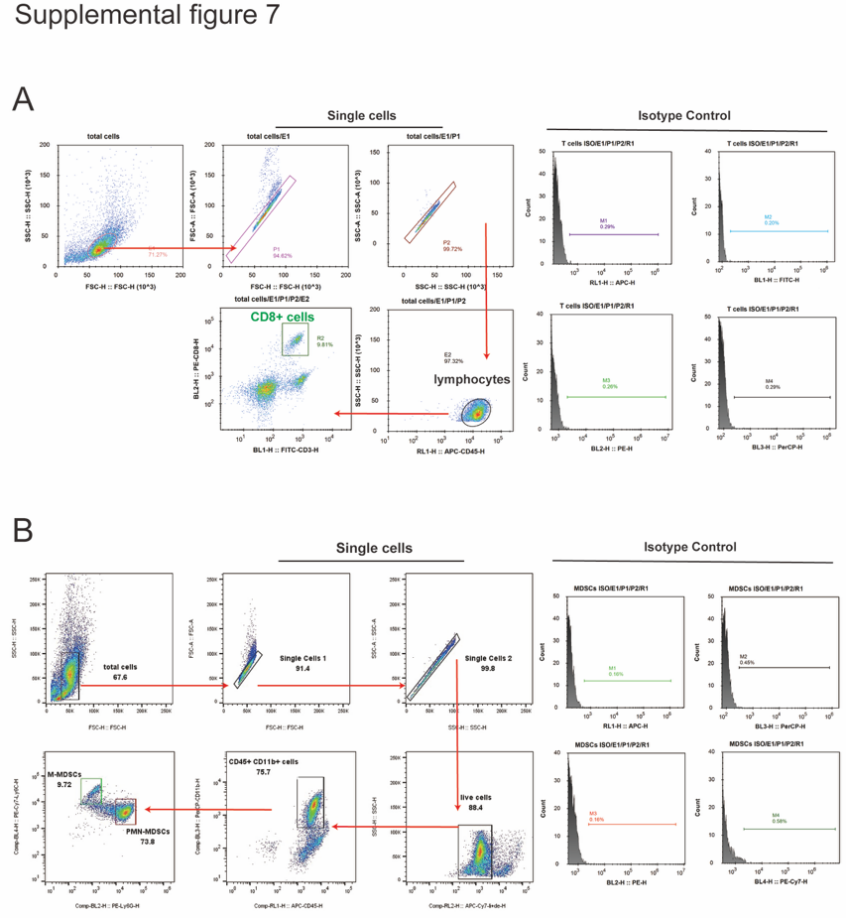


**Supplemental Figure 1. Flow cytometry gating and isotype controls**

(A) T‑cell gating: total cells → singlets → CD45^+^ lymphocytes → CD3^+^CD8^+^ cells; corresponding isotype histograms shown on the right. Representative percentages are annotated.

(B) MDSCs gating: total cells → singlets → live cells → CD45^+^CD11b^+^ → Ly6G^+^ PMN‑MDSCs and Ly6C^+^ M‑MDSCs; matched isotype controls on the right. All gates were optimized using FMO/isotype controls.


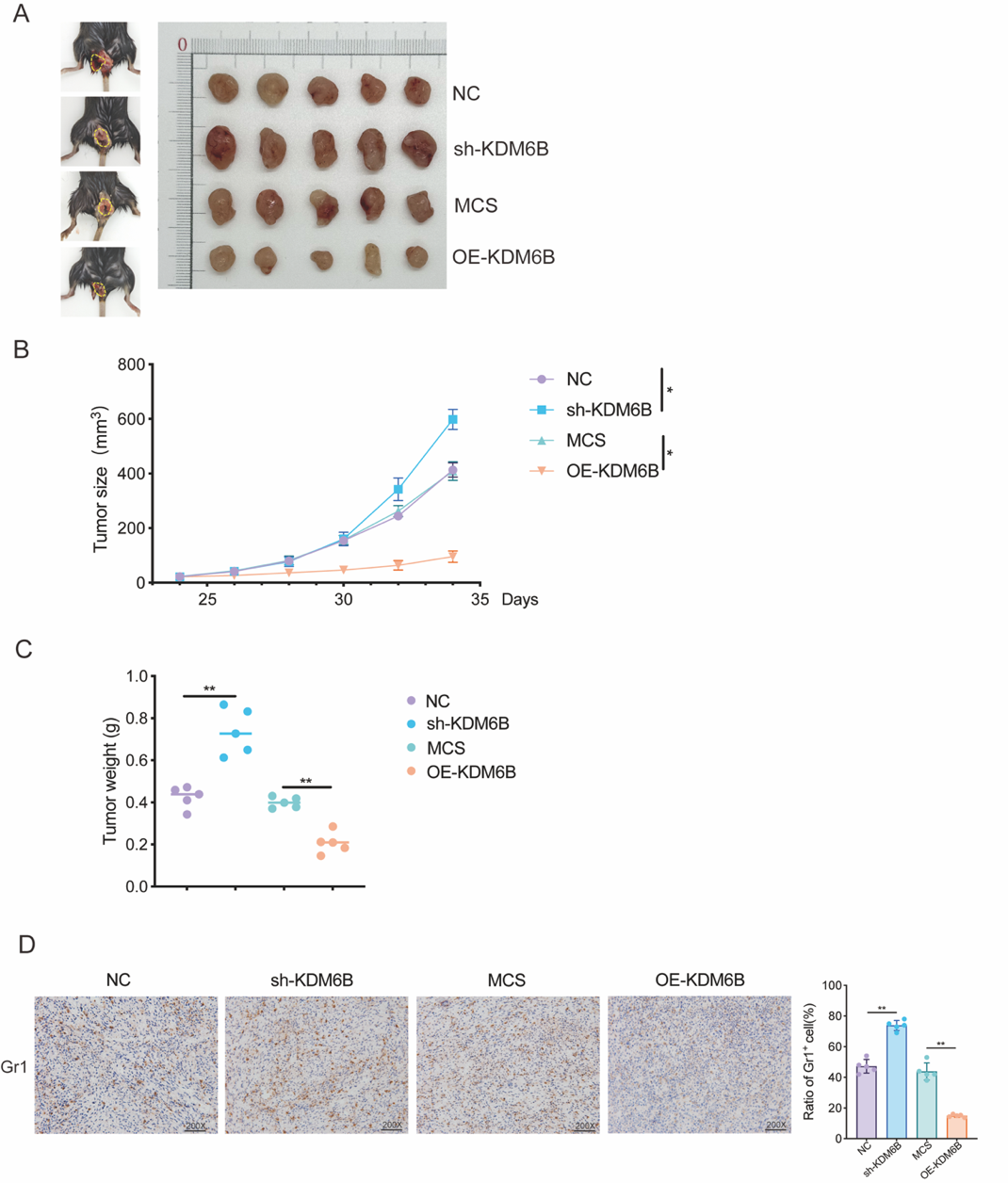


**Supplemental Figure 2. Anti‑tumor role of KDM6B in colorectal cancer xenografts**

(A) Representative in situcutaneous tumor views and excised tumors from control (NC or MCS), KDM6B knockdown (sh‑KDM6B), and KDM6B overexpression (OE‑KDM6B) groups.

(B) Tumor growth curves. Statistics: repeated‑measures two‑way ANOVA with Tukey’s multiple comparisons at the terminal time point; means ± SEM. n=5 per group. * P<0.05.

(C) End‑point tumor weights. Statistics: one‑way ANOVA with Tukey’s post hoc test; means ± SEM. n=5 per group. ** P<0.01.

(D) Representative immunohistochemistry of Gr‑1 in xenograft tumors from NC/MCS controls, KDM6B‑knockdown (sh‑KDM6B), and KDM6B‑overexpression (OE‑KDM6B) groups, with corresponding quantification of Gr‑1^+^ cell proportions. Brown staining indicates Gr‑1^+^ cells; nuclei were counterstained with hematoxylin. Scale bar, 200 μm. Quantification shown as means ± SEM; n = 5 tumors per group. Statistics: one‑way ANOVA with Tukey’s post hoc test. ** P < 0.01.


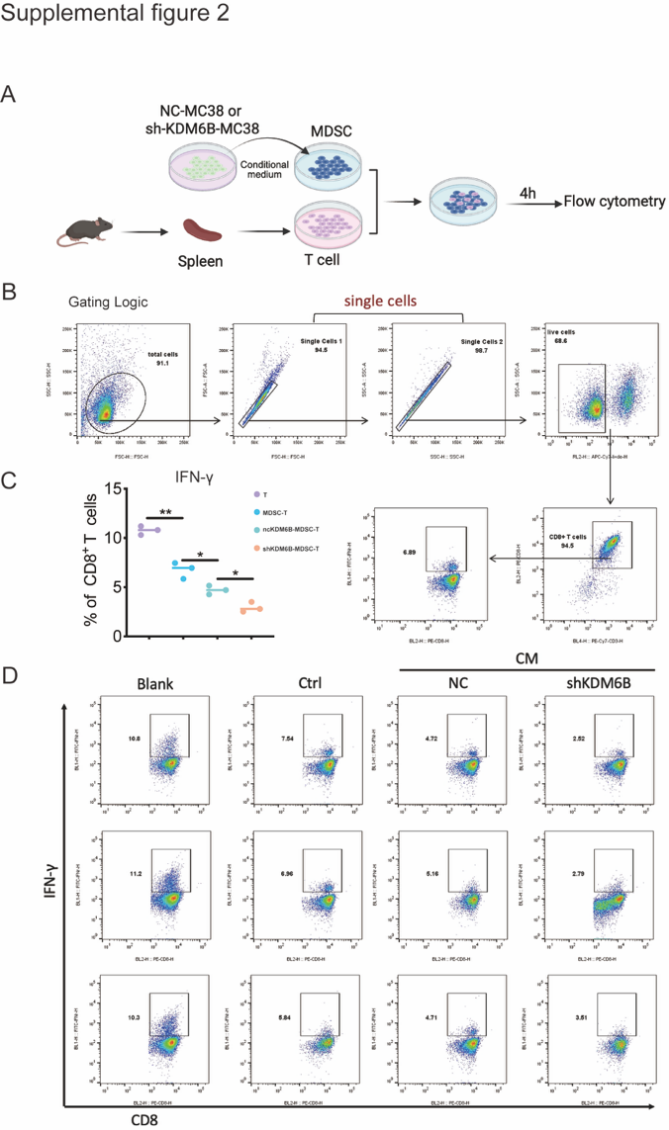


**Supplemental Figure 3.** **KDM6B modulates the suppressive effect of MDSC on CD8^+^ T‑cell IFN‑γ production in vitro**

(A) Experimental workflow: Splenic CD8^+^T cells were isolated from mice and co‑cultured for 4 h with MDSCs generated with conditional medium (CM) from NC‑MC38 or sh‑KDM6B‑MC38 cells, followed by flow cytometric analysis of CD8^+^IFN‑γ^+^ T cells.

(B) Gating strategy: total cells → singlets (FSC/SSC‑H vs ‑W) → live cells (viability dye–negative) → CD3^+^CD8^+^ T cells → IFN‑γ^+^ population. Representative gates are shown on the right.

(C) Quantification of CD8^+^IFN‑γ^+^ T cells across conditions (T alone; MDSC+T; NC‑MDSC+T; shKDM6B‑MDSC+T).

(D) Representative flow plots of CD8 versus IFN‑γ under Blank (T alone), Ctrl (MDSC+T baseline), NC‑CM, and shKDM6B‑CM. Three representative technical replicates are shown per condition.

Dots represent individual samples; lines denote means. Statistics: one‑way ANOVA with Tukey’s multiple comparisons, two‑sided. Data are means ± SEM. n=3 independent biological replicates. Significance symbols: * P<0.05; ** P<0.01, ns=no significance.


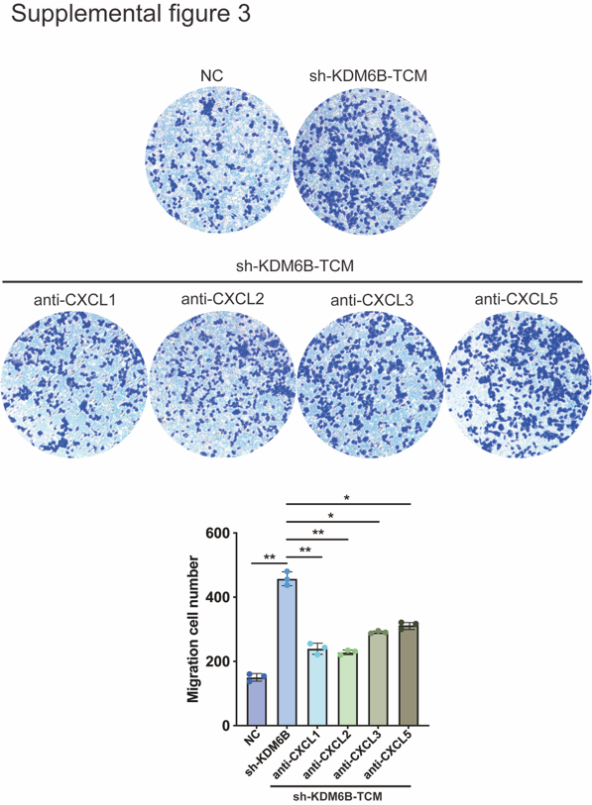


**Supplemental Figure 4. sh‑KDM6B tumor‑conditioned medium (TCM) enhances MDSCs migration; blockade of selected chemokines mitigates the effect**

Top, Representative Transwell images: NC‑TCM, sh‑KDM6B‑TCM, and sh‑KDM6B‑TCM supplemented with neutralizing antibodies against CXCL1/2/3/5.

Bottom, Quantification of migrated cells. Statistics: one‑way ANOVA with Tukey’s multiple comparisons; means ± SEM. n=3 independent experiments. ns, not significant; * P<0.05; ** P<0.01.


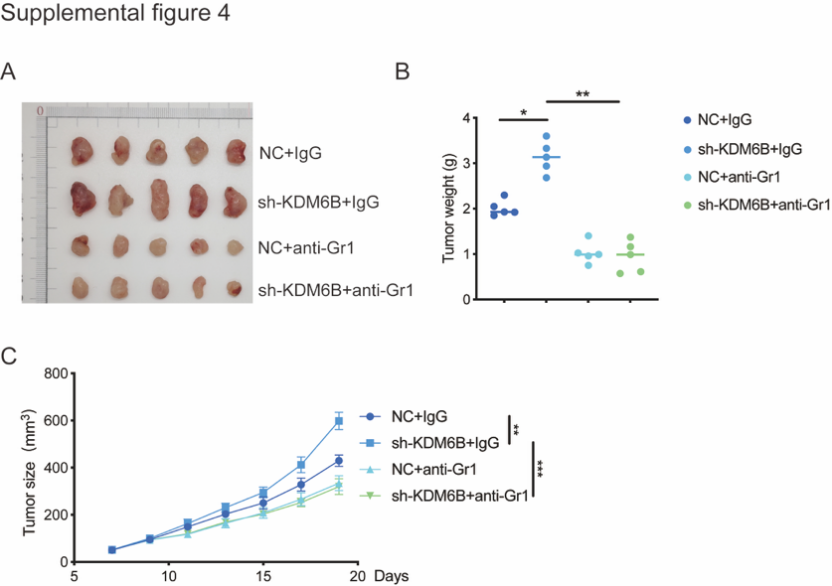


**Supplemental Figure 5. Gr‑1 neutralization attenuates sh‑KDM6B–driven tumor growth**

(A) Representative tumor pictures from mice bearing MC38 control (NC) or KDM6B‑knockdown (sh‑KDM6B) tumors treated with IgG or anti‑Gr1.

(B) End‑point tumor weights. Each dot is one mouse; short bars indicate means. Statistics: one‑way ANOVA with Tukey’s post hoc test, two‑sided; means ± SEM. n=5 per group.

(C) Tumor growth curves over time. Statistics: repeated‑measures two‑way ANOVA (group × time); Tukey’s multiple comparisons at the terminal time point; means ± SEM. n=5 per group. * P<0.05; ** P<0.01.


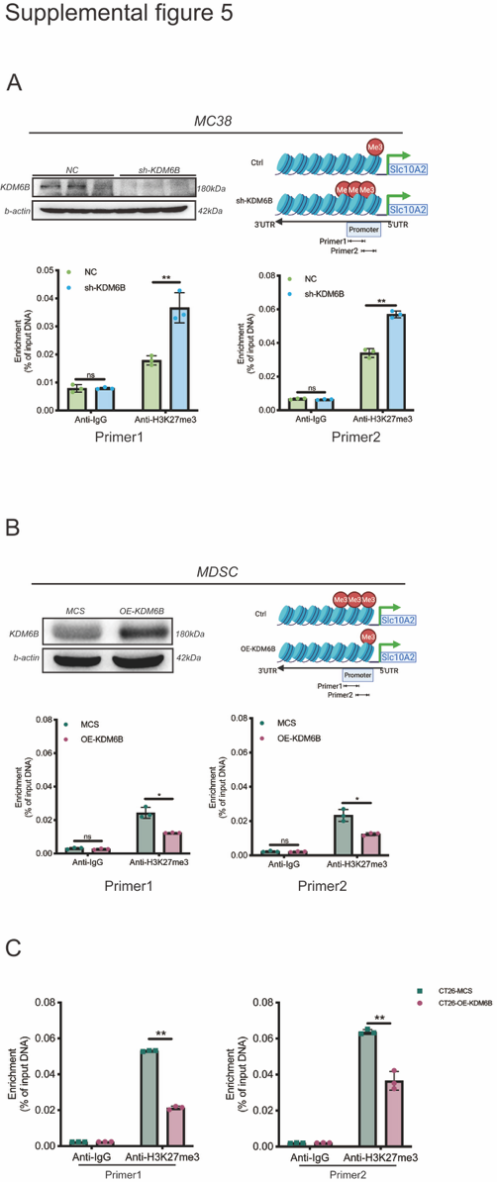


**Supplemental Figure 6.** **KDM6B directly regulates SLC10A2 transcription via H3K27me3 demethylation in tumor and myeloid cells**.

(A) ChIP–qPCR analysis of H3K27me3 occupancy at the SLC10A2 promoter in MC38 cells transfected with control (NC) or KDM6B siRNA. KDM6B knockdown markedly increased H3K27me3 enrichment, indicating that KDM6B directly promotes SLC10A2 transcription through H3K27me3 demethylation.

(B) ChIP–qPCR analysis of H3K27me3 levels at the SLC10A2 promoter in MDSC overexpressing KDM6B or control vector. KDM6B overexpression significantly reduced H3K27me3 enrichment, demonstrating that this regulatory axis operates in both tumor epithelial and myeloid cells.

(C) ChIP‑qPCR for H3K27me3 at two SLC10A2 promoter regions (Primer1/Primer2) in CT26‑MCS and CT26‑OE‑KDM6B cells.

Data represent mean ± SEM from three independent experiments. Statistical significance was assessed by two-tailed unpaired Student’s t-test; Quantification shown as means ± SEM. Significance symbols: * P<0.05; ** P<0.01, ns=no significance.


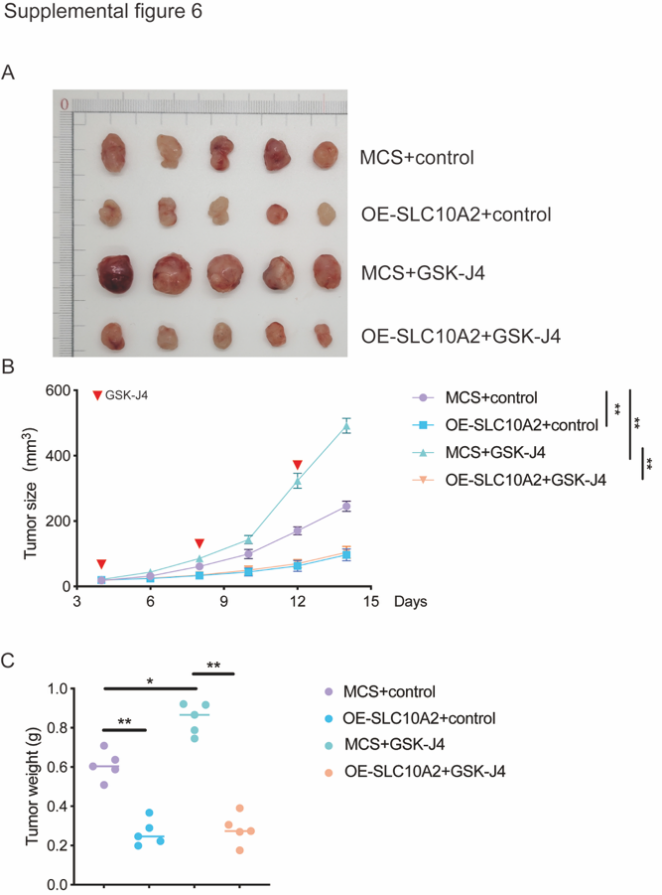


**Supplemental Figure 7.** **Overexpression of SLC10A2 opposes GSK‑J4–driven tumor growth**

(A) Representative tumor images from mice bearing vector control (MCS) or SLC10A2‑overexpressing (OE‑SLC10A2) tumors treated with vehicle or GSK‑J4. OE‑SLC10A2 reduces tumor size relative to MCS, whereas GSK‑J4 increases tumor size; OE‑SLC10A2 mitigates GSK‑J4–induced tumor growth.

(B) Tumor growth curves over time; red arrowheads indicate dosing time points. Statistics: repeated‑measures two‑way ANOVA with Tukey’s multiple comparisons at the terminal time point; means ± SEM. n=5 per group.

(C) End‑point tumor weights. Statistics: one‑way ANOVA with Tukey’s post hoc test; means ± SEM. n=5 per group. Significance symbols: * P<0.05; ** P<0.01.
